# Functional annotation and meta-analysis of maize transcriptomes reveal genes involved in biotic and abiotic stress

**DOI:** 10.1186/s12864-024-10443-7

**Published:** 2024-05-30

**Authors:** Rita K Hayford, Olivia C Haley, Ethalinda K Cannon, John L Portwood, Jack M Gardiner, Carson M Andorf, Margaret R Woodhouse

**Affiliations:** 1 Corn Insects and Crop Genetics Research Unit, USDA-ARS, Ames, IA 50011 USA; 2 Division of Animal Sciences, University of Missouri, Columbia, MO 65211 USA; 3 Department of Computer Science, Iowa State University, Ames, IA 50011 USA

**Keywords:** Differentially expressed genes, Maize, RNA-Sequencing, Transcription factors, Gene Ontology, Abiotic stress, Biotic stress

## Abstract

**Background:**

Environmental stress factors, such as biotic and abiotic stress, are becoming more common due to climate variability, significantly affecting global maize yield. Transcriptome profiling studies provide insights into the molecular mechanisms underlying stress response in maize, though the functions of many genes are still unknown. To enhance the functional annotation of maize-specific genes, MaizeGDB has outlined a data-driven approach with an emphasis on identifying genes and traits related to biotic and abiotic stress.

**Results:**

We mapped high-quality RNA-Seq expression reads from 24 different publicly available datasets (17 abiotic and seven biotic studies) generated from the B73 cultivar to the recent version of the reference genome B73 (B73v5) and deduced stress-related functional annotation of maize gene models. We conducted a robust meta-analysis of the transcriptome profiles from the datasets to identify maize loci responsive to stress, identifying 3,230 differentially expressed genes (DEGs): 2,555 DEGs regulated in response to abiotic stress, 408 DEGs regulated during biotic stress, and 267 common DEGs (co-DEGs) that overlap between abiotic and biotic stress. We discovered hub genes from network analyses, and among the hub genes of the co-DEGs we identified a putative NAC domain transcription factor superfamily protein (*Zm00001eb369060*) IDP275, which previously responded to herbivory and drought stress. IDP275 was up-regulated in our analysis in response to eight different abiotic and four different biotic stresses. A gene set enrichment and pathway analysis of hub genes of the co-DEGs revealed hormone-mediated signaling processes and phenylpropanoid biosynthesis pathways, respectively. Using phylostratigraphic analysis, we also demonstrated how abiotic and biotic stress genes differentially evolve to adapt to changing environments.

**Conclusions:**

These results will help facilitate the functional annotation of multiple stress response gene models and annotation in maize. Data can be accessed and downloaded at the Maize Genetics and Genomics Database (MaizeGDB).

**Supplementary Information:**

The online version contains supplementary material available at 10.1186/s12864-024-10443-7.

## Background

Maize (*Zea mays* ssp. mays L.) is one of the most widely grown crops across the globe. The importance of maize goes beyond food and animal feed; it is currently used as a biofuel source, and is also an important genetic model plant [1–4]. The growth and development of maize is highly dependent on suitable climatic and soil conditions [3, 5, 6]. However, major abiotic stress factors such as drought, salinity, and extreme temperatures, and biotic stresses including fungal, bacterial, and viral pathogens, adversely affect maize production [6]. Abiotic stress can cause a 54reduction of over 50% yield of global crop production, with drought, heat, salinity and cold stress the main threat to maize production in major maize cultivation regions [4, 7–9]. Drought and heat stress cause up to 40% of global yield loss of maize [10], and biotic stress leads to a yearly loss of approximately 10% of maize yield worldwide [6]. One of the biotic factors that affect crop growth is herbivory of pest insects, with arthropods making up 6–19% of herbivore attack [10]. Climate change potentially increases plant exposure to these environmental stressors, which could increase in occurrence, intensity, and complexity due to global warming, climate variability, and industrial pollution. For example, climate change can directly impact stress factors involved in host plants and pathogens and affect temperature changes, increasing biotic and abiotic stress, respectively [11, 12].

Because of these stresses, it is necessary to implement strategies to develop maize varieties with improved yield and climate resilience. Omics approaches such as genomics, transcriptomics, proteomics, and metabolomics have been integrated with maize breeding strategies. For instance, the availability of transcriptome profiling technologies has been used to understand the complexity of gene expression during plant development and under stress conditions [13]. High-throughput next-generation sequencing approaches like RNA-Seq have recently been used to analyze the transcriptomes of different crops under different stress conditions [13–15]. Transcriptome profiling has generally been used to analyze the transcriptomes of crops such as Arabidopsis, maize, wheat, rice, and soybean [16], and significant progress has been made in understanding the molecular mechanisms of plant responses to abiotic stress factors [3].

Plants produce various reactions at the molecular level in response to stress. Transcription factors (TFs) have been identified to play critical roles in providing stress tolerance to different stresses [3, 17], such as abscisic acid synthesis response. In the last few years, many transcriptome studies have discovered various pathogen responses in different maize lines [18]. The advances in high-throughput sequencing technologies have led to increased numbers of proteomic data, but in many cases, their function has to be determined. Therefore, accurately annotating maize proteins would be helpful for further downstream experiments [19, 20].

Plant stress response can be complex; some response mechanisms can evolve either at the biochemical or physiological level, providing a source for gene adaptability to environmental changes. Phylostratigraphic analysis has become a promising tool that helps to determine the time of occurrence of genes to assess their age and link the ages of the genes with their functional role [21]. A phylostratigraphic analysis of different stress response genes can assist researchers in understanding the evolutionary trajectory of various plant stress responses.

Here we mapped 24 publicly available, high-quality RNA-Seq read datasets o the recent version of the reference genome B73 (B73v5) [22] and deduced stress-related functional annotation of gene models using an in-house developed pipeline. We analyzed the datasets to determine specific and common differentially expressed genes (DEGs) for biotic and abiotic stress. Gene ontology or enrichment analysis of the DEGs was performed to gain knowledge regarding the biological processes of the stress genes. Functional analysis revealed key genes, pathways and annotations for genes involved in different stress. Furthermore, we identified stress genes encoding maize transcription factors, and we constructed a network analysis that revealed a hub of genes important for stress response. Finally, we analyzed DEGs from this study associated with the different types of stress (abiotic, biotic, co-DEGs) using a phylostratigraphic approach. For the phylostratigraphic analysis, we observed that abiotic stress genes fell under similar, more ancient phylostratigraphic categories, suggesting that abiotic stress adaptation is more conserved. In contrast, biotic stress genes have a more diverse phylostratigraphic profile, suggesting that biotic stress gene evolution is more dependent on the host genes involved within more diverse environments. The data and results from this study will help annotate maize-responsive genes, thereby supporting strategies for improving maize varieties.

## Results

### Overview of RNA-Seq datasets

**Fig. 1:**
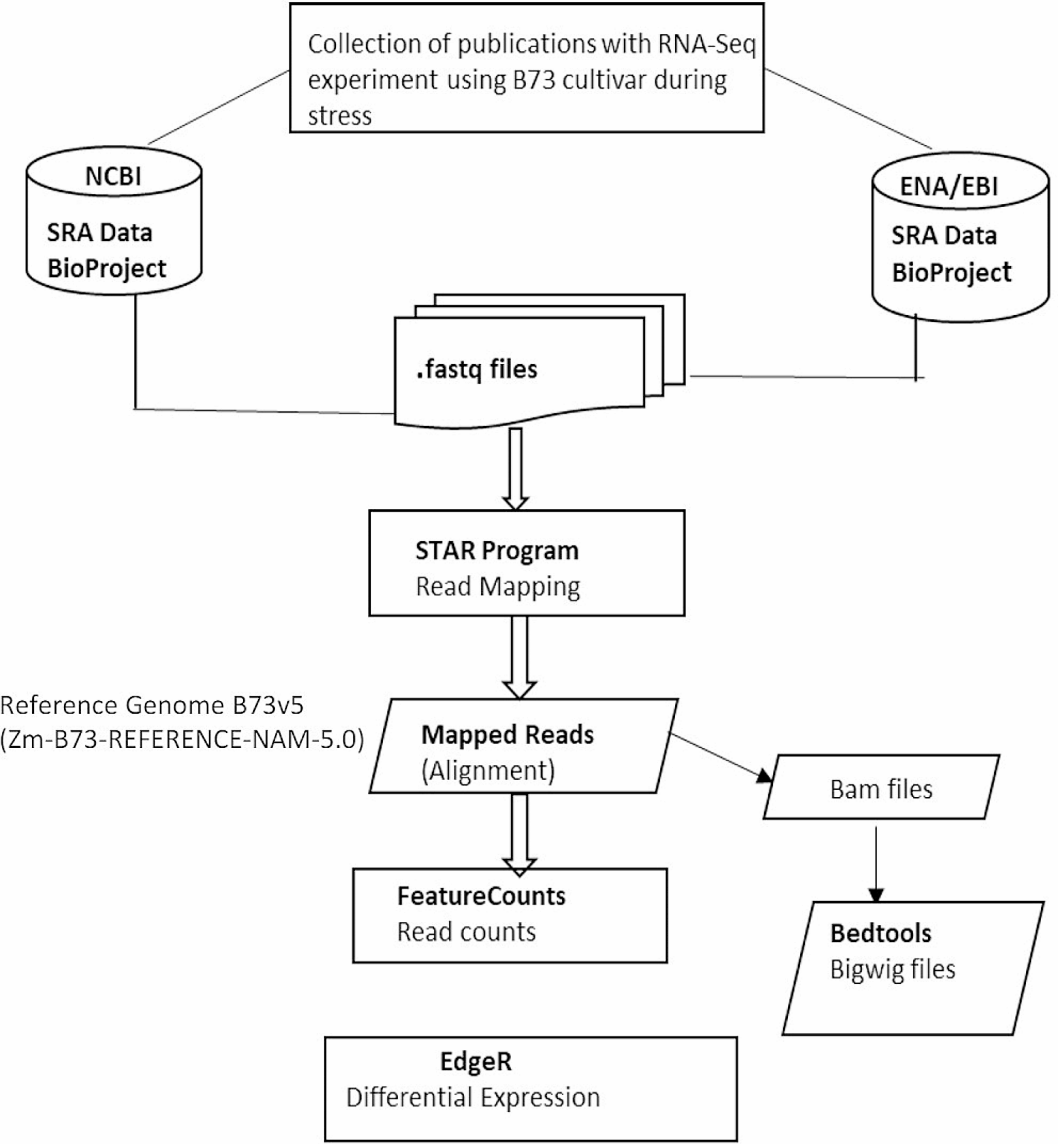
A Pipeline to map high-quality RNA-Seq data in Maize

We retrieved 24 RNA-Seq datasets with high-quality reads related to biotic and abiotic stress generated from the B73 cultivar (Methods). All 24 RNA-Seq datasets were mapped to earlier versions of B73 using our pipeline (Fig. 1). The dataset is made up of 17 abiotic and 7 biotic stress studies (Additional file 1). The publication years of the datasets range from 2014 to 2022. The RNA-Seq studies collected for this study captured various types of abiotic stress factors, including: drought, heat, cold, salinity, waterlogging, nitrogen, cadmium, phosphate, nitrate, ammonium and elevated ozone (UV). The biotic datasets included samples exposed to *Cercospora Zeina* (causal agent of gray leaf spot), *Fusarium graminearum* (causal agent of Gibberella stalk rot (GSR)), *Fusarium venenatum*, *Colletotrichum graminearum*, Sugarcane Mosaic Virus (SCVM), or Mites herbivores. The detailed information of the RNA-Seq data used in this study is summarized in Additional file 1.

### Differential expression analysis

**Fig. 2:**
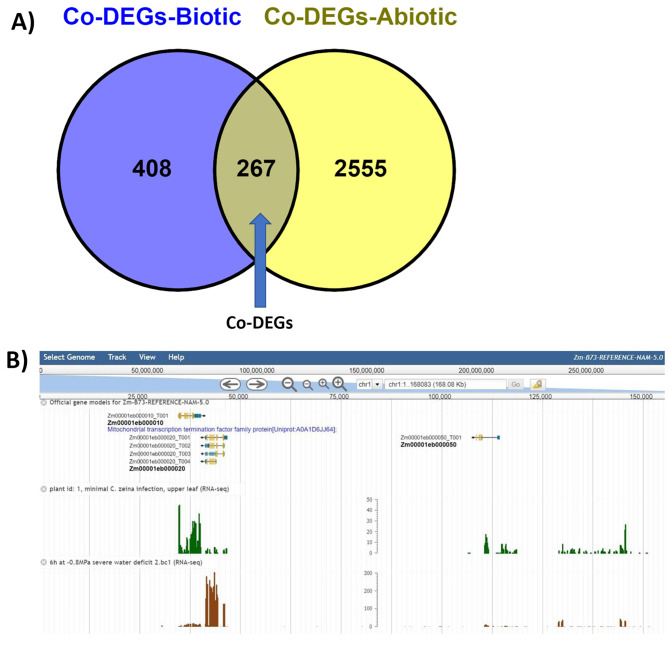
Differentially expressed genes (DEGs) in the 24 experiments with B73 imposed with either biotic or abiotic stress. **(A)** Venn diagram showing DEGs specifically expressed during abiotic (common abiotic DEGs; co-DEGs-Abiotic) and the biotic (common biotic DEGs; co-DEGs-Biotic) and the overlap of DEGs among the stresses **(B)** Example of stress-responsive gene models available as genome browser tracks hosted by MaizeGDB

A fold-change value of [|log2(fold change) | ≥ 1, *p*-value < 0.05] was used to determine a significant stress response for each candidate gene model. The number of genes specifically expressed in each treatment per experiment is indicated in Additional file 1. Pairwise comparisons between stress-treated and control-treated samples in each experiment were applied to determine the DEGs. The number of DEGs varied depending on the type of stress, and DEGs were separated into the number of up-regulated or down-regulated DEGs for each treatment within an experiment. For example, the RNA-Seq experiment with project accession PRJNA335771 describes B73 subjected to either salinity, drought, heat and cold stress conditions. Each of the abiotic stress sample was compared with the control to identify DEGs ([|log2(fold change) | ≥ 1, *p*-value < 0.05]). From the pairwise comparisons with the control samples for the above experiment, we identified 5,379 (2,630 up- and 2,749 down-regulated) DEGs in the salinity stress sample, 2,840 (1,184 up- and 1,655 down-regulated) in the cold stress sample, 3,143 (1,540 up- and 1,603 down-regulated) in the drought sample and 4,645 (2,451 up- and 2,194 down-regulated) in the heat stress sample. In another example of B73 infected with SCVM- (project accession PRJNA846583), we identified 582 DEGs (348 up- and 234 down-regulated genes) for pairwise comparison between inoculated and mock-inoculated samples. The DEGs for each stress condition of all the datasets were determined and integrated. Possible intersections of all DEGs from the experiments were identified as 3,230 DEGs. Among a total of 3,230 DEGs identified from all the 24 different RNA-Seq datasets, 2,555 and 408 DEGs were generally expressed during abiotic and biotic stress respectively, with 267 DEGs overlapping both stress types (referred to here as common DEGs or co-DEGs) shown in Fig. 2A. An example of the expression of a stress-responsive data set hosted by MaizeGDB on JBrowse is shown in Fig. 2B.

#### Abiotic stress DEGs

From the differential expression analysis, many more genes (2,555) were identified in response to the abiotic stress compared to biotic stress (Fig. 2A). The lists of all the abiotic stress-responsive genes and their description obtained from MaizeMine have been indicated in Additional file 2. These genes are common among all the abiotic stresses and they include genes encoding late embryogenesis abundant (LEA) proteins, heat shock proteins (HSPs), other chaperones, and ion transporters. We identified 55 genes encoding HSPs as chaperones in response to abiotic stress. The Late Embryogenesis Abundance (LEA) proteins are known to be involved in plant response to abiotic stresses such as drought, cold, and salinity. From our analysis, LEA proteins were only found in the abiotic DEGs. Conversely, HSPs were associated with abiotic, biotic and overlapping DEGs.

#### Biotic stress DEGs

From the differential expression analysis, 408 DEGs were identified in response to biotic stress (Fig. 2A). The lists of biotic stress-responsive genes and their descriptions are indicated in Additional file 2. Similar to the abiotic DEGs, the list of biotic DEGs genes are associated with descriptions common to biotic stresses, including PR5 (*Zm00001eb032580*), CHIA-chitinase (*Zm00001eb078730*), Disease resistance protein RPM1(*Zm00001eb226700*), Protein kinase domain-containing protein (*Zm00001eb170460*), and Wall-associated receptor kinase-like 20 (*Zm00001eb177830*). Significant candidate DEGs include potential PRR genes such as WAK-RLK (*Zm00001eb177830)*, known to confer pathogen resistance in maize; two LRR-RLK genes (*Zm00001eb293660* and *Zm00001eb153630*); and two chitinase genes (*Zm00001eb167720* and *Zm00001eb1677).* In addition, we identified two genes encoding HSPs in response to biotic stress which are *Zm00001eb012470* (Heat shock cognate 70 kDa protein 2) and *Zm00001eb314890* (Heat stress transcription factor B-2b).

#### Co-DEGs

Based on the overlap of DEGs in biotic and abiotic stresses, 267 genes were observed as being in both sets (Fig. 2A). DEGs identified as a result of the expression responses to the various biotic and abiotic stresses are listed in Additional file 2, and they include Glutathione transferases, Pathogenesis-related proteins, Chitinase, and Aquaporins. Similar to biotic stress response, we identified two genes encoding HSPs among the co-DEG list. These are *Zm00001eb109480* (Putative Heat stress transcription factor A-2c) and *Zm00001eb198620* (Heat stress transcription factor B-2b).

### Functional classification of DEGs

#### Functional classification of abiotic DEGs

**Fig. 3:**
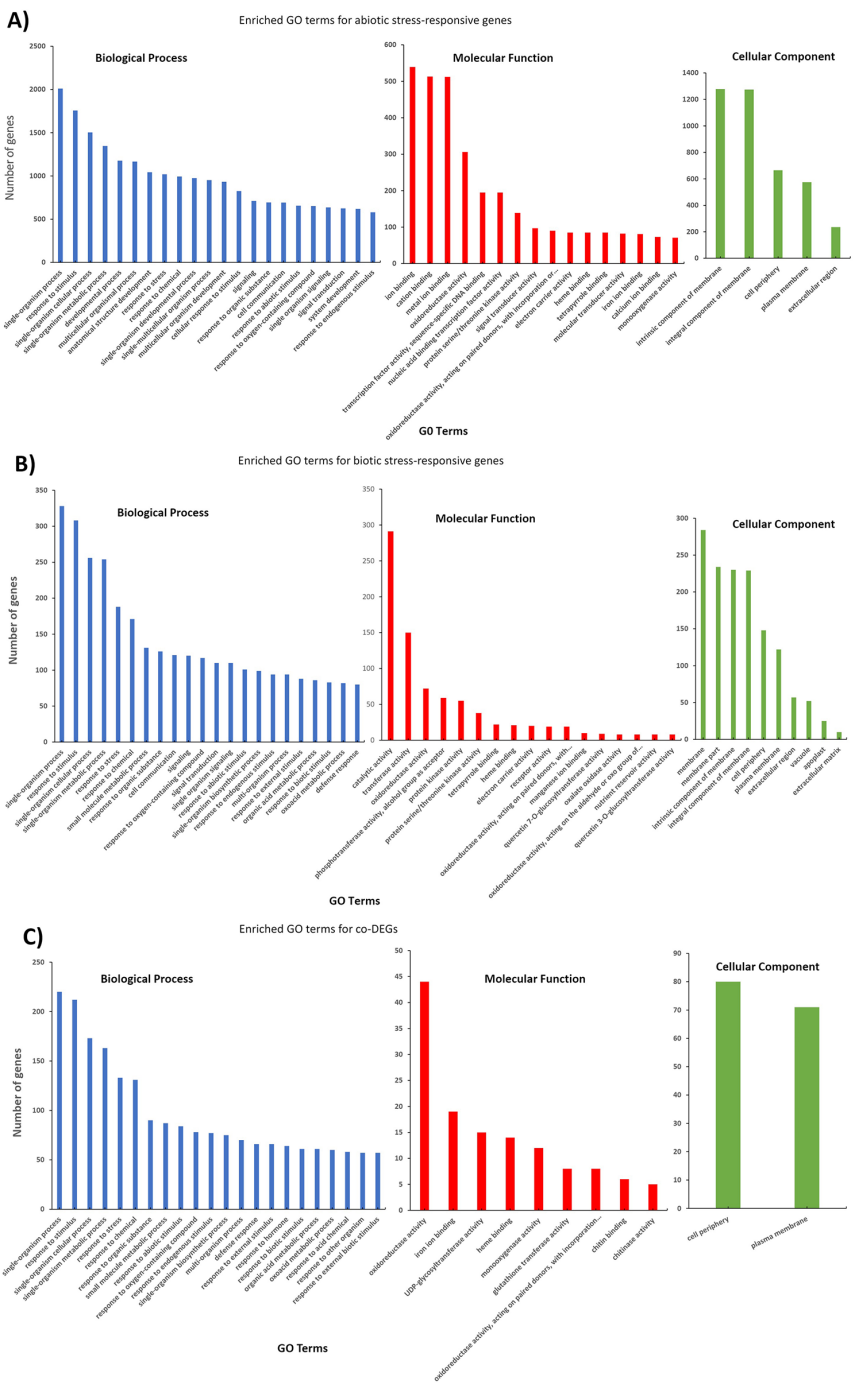
(**A**) The Gene Ontology (GO) terms enriched by abiotic stress-responsive genes. (**B**) GO terms enriched by biotic stress-responsive genes. (**C**) GO terms enriched by co-DEGs. The GO terms are in the three GO domains (biological process, molecular function and cellular component). All the GO terms in Fig. 3 and Additional file 3 are significantly enriched (*p* < 0.05) using Fisher’s exact test and Bonferroni multi-test adjustment from AgriGO v2.0 software. The number of genes enriched in each term were plotted against the GO term. More details in Additional file 3

To better understand the expression changes that occur in response to different environmental stresses, the enriched GO terms associated with the abiotic DEGs, a GO analysis was performed to determine the biological functions of the identified stress-responsive genes using AgriGO (Du et al. 2010, Tian et al. 2017) (Fig. 3A). The GO annotations of this group showed a number of significantly enriched terms associated with stress response, particularly “response to oxygen-containing compound”. Other significant enriched terms in the biological process category include: “response to abiotic stimulus”, “response to chemical”, “response to jasmonic acid” and “response to abscisic acid”. Strikingly, many abiotic stress-responsive genes were enriched in the non-stress specific GO term “developmental process”. Within the molecular function category, the most significantly enriched terms were “transcription factor activity”, “sequence-specific DNA binding”, and “nucleic acid binding transcription factor activity”. Other significantly enriched molecular function GO terms were “cation binding”, “oxidoreductase activity”, and “ion binding”. For the cellular component category, the most significantly enriched terms were “plasma membrane” and “cell periphery” (Additional file 3).

#### Functional classification of biotic DEGs

As shown in Fig. 3B and Additional file 3, the most significantly enriched GO -biological processes for the biotic DEGs is “response to oxygen-containing compound”. Other enriched terms in the biological processes are “response to chemical”, “response to stimulus”, “response to biotic stimulus”, “response to chitin”, and “secondary metabolic process”. Within the molecular function category, the most significantly enriched terms are “oxalate oxidase activity”, “oxidoreductase activity acting on the aldehyde or oxo group of donors”, “oxygen as acceptor”, and “protein serine/threonine kinase activity”. Otherwise, most of the biotic DEGs were enriched in catalytic and transferase activity. For the cellular component category, the most significantly enriched terms were “plasma membrane” and “cell periphery” (Additional file 3).

#### Functional classification of co-DEGs

In the biological enrichment process of co-DEGs (Fig. 3C), we found “response to chemical” as the most significant term. Similar to the biotic and abiotic stress DEGs, many of the co-DEGs were enriched in the biological process terms “single-organism process”, “response to stimulus”, “single-organism cellular process”, “single-organism metabolic process”, and “response to stress”. In the molecular function category, the most significant enriched terms for the co-DEGs are “iron ion binding” and “chitin binding”. However, most of the co-DEGs for molecular function were enriched in oxidoreductase and UDP-glycosyltransferase activity. Only two significantly enriched terms were identified for this group of genes, “plasma membrane” and “cell periphery”.

### Kyoto encyclopedia of genes and genomes (KEGG) pathway analysis

#### KEGG pathway analysis of abiotic stress DEGs

**Fig. 4:**
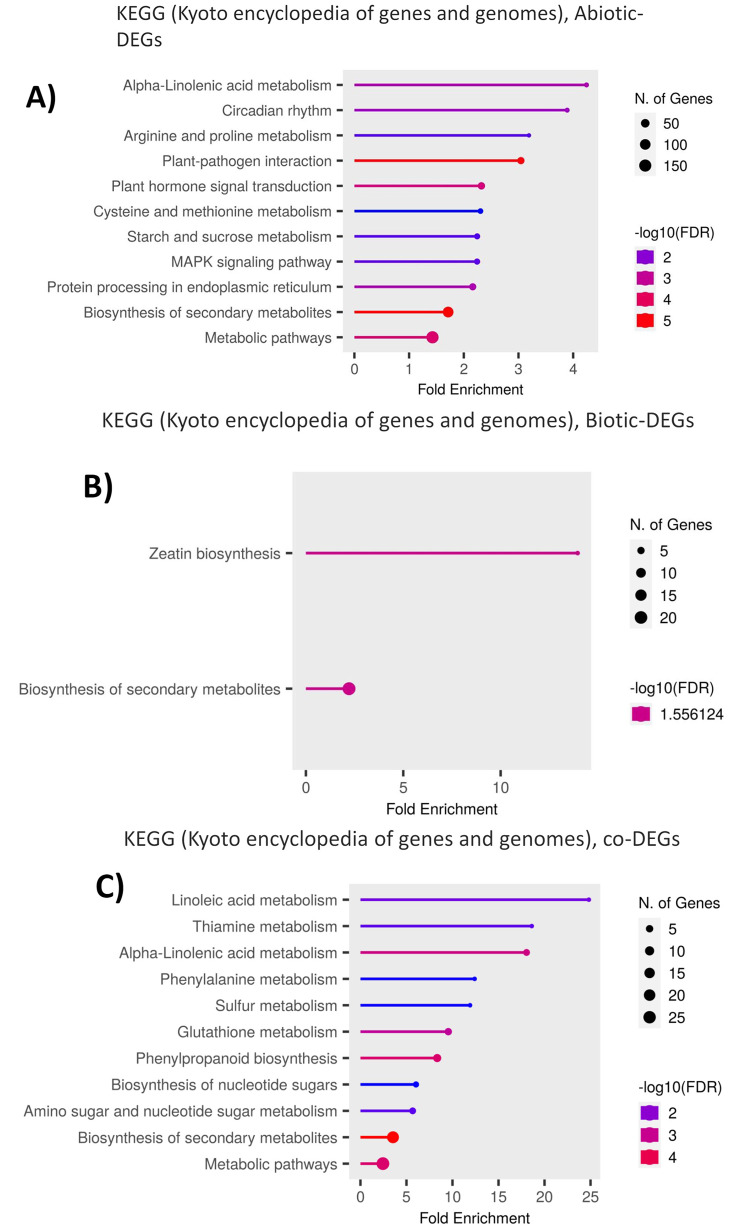
Kyoto Encyclopedia of Genes and Genomes (KEGG) analysis of (**A**) abiotic stress-responsive genes. (**B**) biotic stress responsive genes and (C) Co-DEGs of maize. The enrichment FDR was set to a cutoff of 0.05

To further explore the biological pathways of the DEGs involved in maize abiotic stress, we performed KEGG enrichment analysis using ShinyGO (Methods). The enriched pathways associated with the annotated abiotic stress-responsive genes have been illustrated in Fig. 4A. Here, 151 and 102 DEGs were mapped to the *metabolic* and *biosynthesis of secondary metabolites* pathways respectively. Other enriched pathways for this group of DEGs are *plant hormone signal transduction*, *MAPK signaling pathway*, and *alpha-linolenic acid metabolism*. Other abiotic DEGs that mapped to each pathway are listed in Additional file 4.

#### KEGG pathway analysis of biotic stress DEGs

The enriched pathways associated with the annotated biotic stress-responsive genes are illustrated in Fig. 4B. Only two pathways were significant: “*biosynthesis of secondary metabolites*” (21 biotic DEGs mapped), and “*Zeatin biosynthesis*” (three biotic DEGs mapped) (Additional file 4).

#### KEGG pathway analysis of co-DEGs

The enriched pathways associated with the annotated co-DEGs have been illustrated in Fig. 4C. Similar to the abiotic stress-responsive genes, most of the co-DEGs are significantly enriched in the *Metabolic* and *Biosynthesis of secondary metabolites pathway*. Interestingly, *phenylpropanoid biosynthesis* was identified as another significantly enriched pathway for the overlapping genes in biotic and abiotic stress. The lists of genes for each of the pathways can be found in Additional file 4.

### Transcription factor analysis

#### TF analysis of abiotic DEGs

The abiotic stress DEGs encoding TFs were obtained as described in Methods. A total of 352 TFs were enriched from the 2555 DEGs (Additional file 5). Percent TF enrichment in the DEGs are: WRKY (4.5%), MYB (9%), NAC (6.3%), bHLH (8.2%), EREB (12.5%), bZIP (3.4%), and GRAS (2.5%). Further analysis of the abiotic TFs using the expression data from project accession PRJNA335771 (containing drought, heat, cold and salinity DEGs) showed that the major TFs (WRKY, NAC, EREB, bZIP, and bHLH) are associated with the regulation of abiotic stress response in maize. There were more up-regulated versus down-regulated TFs in response to the abiotic stresses considered here (Table 1). Interestingly, we found that a higher proportion of the DEGs encoding EREB, bHLH, NAC, WRKY, bZIP, and MYB TF families were enriched in DEGs associated with salinity stress. We also found that 6 NAC TFs were down-regulated during drought stress compared to 2, 1,4 down-regulated genes encoding NAC TF in response to salt, cold and heat stress respectively (Table 1).

**Table 1:** Number of differentially expressed TFs in response to abiotic stress response

	Stress type										
TF family	Salinity			Cold			Heat		Drought		
Regulation	Up	Down		Up	Down		Up	Down	Up	Down	
EREB	37		7	23		3	17	4	17		6
bHLH	22		4	6		-	5	4	4		5
MYB	16		8	6		1	7	3	8		6
NAC	18		2	6		1	3	4	2		6
WRKY	13		2	8		1	2	4	1		1
bZIP	6		4	-		-	5	1	4		2

Notably, our analysis revealed an up-regulation of *ZmWRKY40* (*Zm00001eb149570*) in response to salinity and cold stress. Furthermore, eight TFs from our analysis including *ZmMYBR103* (*Zm00001eb035050*), *ZmMYB2* (*Zm00001eb278680*), *ZmWRKY81* (*Zm00001eb149550*), *ZmNac49* (*Zm00001eb062170*), *ZmbHLH150* (*Zm00001eb314810*), *ZmEREB205* (*Zm00001eb430640*), *ZmEREB83* (*Zm00001eb369560*), and *ZmEREB204* (*Zm00001eb042240*) were induced during drought, cold, heat and salinity stress. All eight TFs were induced in all four stresses (drought, cold, heat and salinity stress) mentioned above. The above-mentioned results suggest the critical role of the TFs in multiple abiotic stress response in maize. Moreover, a number of GATA TFs (*ZmGAT12*, *ZmGATA14*, *ZmGATA34*) which were recently characterized in maize in response to abiotic and biotic stresses were regulated during abiotic stress in our list.

#### TF analysis of biotic DEGs

We were able to associate 28 TFs with the 408 biotic DEGs as described in Methods (details in Additional file 5). Interestingly, many of the TFs from the biotic DEGs belong to the WRKY family, which is seven out of the 28 TFs (∼ 32%). The fraction of the other TFs represented are NAC (3.5%), bHLH (10.7%), EREB (18%), MYB (3.5%). Analysis of the biotic TFs using expression data from biotic experiments revealed that a total of 15 WRKY genes associated Mite herbivore infections were up-regulated. All 15 WRKY TFs induced by herbivory were up-regulated during separate infections from the two species; the generalist twospotted spider mite (*Tetranychus urticae*, TSM) and the specialist Banks grass mite (*Oligonychus pratensis*, BGM) classified under Mite herbivore (Table 2). We found that a higher proportion of the DEGs encoding EREB, bHLH, NAC, and MYB TF families were enriched in DEGs associated with the infection of Mite herbivores. It is worth mentioning that although a large proportion of the DEGs encoding EREB were in response to Mites herbivore, there was a relatively large amount of EREB TFs that were down-regulated in response to *F*. *graminearum.* This may indicate the negative role of EREB TFs during maize infection with.

*F*. *graminearum* (GSR disease). These three NAC genes, *ZmNAC20* (*Zm00001eb288360*), *ZmNAC25* (*Zm00001eb405590*), and *ZmNAC40* (*Zm00001eb183190*) were induced in maize during *F. graminearum*, *F*. *venenatum*, and Mites herbivore infection. Further analysis showed that the above three infections significantly induced *ZmEREB2* (*Zm00001eb429870), ZmEREB198* (*Zm00001eb074930*), and *ZmMYBR105* (*Zm00001eb081280*). These results suggest a critical role for these TFs in plant defense.

**Table 2:** Number of differentially expressed TFs in response to biotic stress response

	Stress type										
TF family	Mite herbivores (both TSM &BGM)			Sugar mosaic virus (SCMV)			Fusarium venenatum		Fusarium graminearum (GSR)		
Regulation	Up	Down		Up	Down		Up	Down	Up	Down	
EREB	33		3	-		2	9	1	4		18
bHLH	19		3	-		-	1	4	2		3
MYB	19		6	-		1	7	2	10		2
NAC	14		2	-		-	9	-	4		-
WRKY	15		-	-		2	7	1	1		1

#### TF analysis of co-DEGs

Out of the 267 co-DEGs, we obtained 22 genes that were encoded by TFs (details in Additional file 4). Similar to abiotic and biotic DEGs, these TF families were represented by WRKY (19.3%), MYB (3.2%), NAC (12.9%), bHLH (3.2%), EREB (16%%), and bZIP (6.5%). Among the TFs of the co-DEGs we identified *ZmNAC126* (*Zm00001eb093650*) and found it to be up-regulated in response to the following stresses: heat, drought, salinity, cadmium, phosphorus, *F. graminearum*, F, *venenatum*, SCVM, and *Colletotrichum graminocola.*

### Network analysis

#### Interaction network of abiotic stress genes and phylostratigraphic analysis

**Fig. 5:**
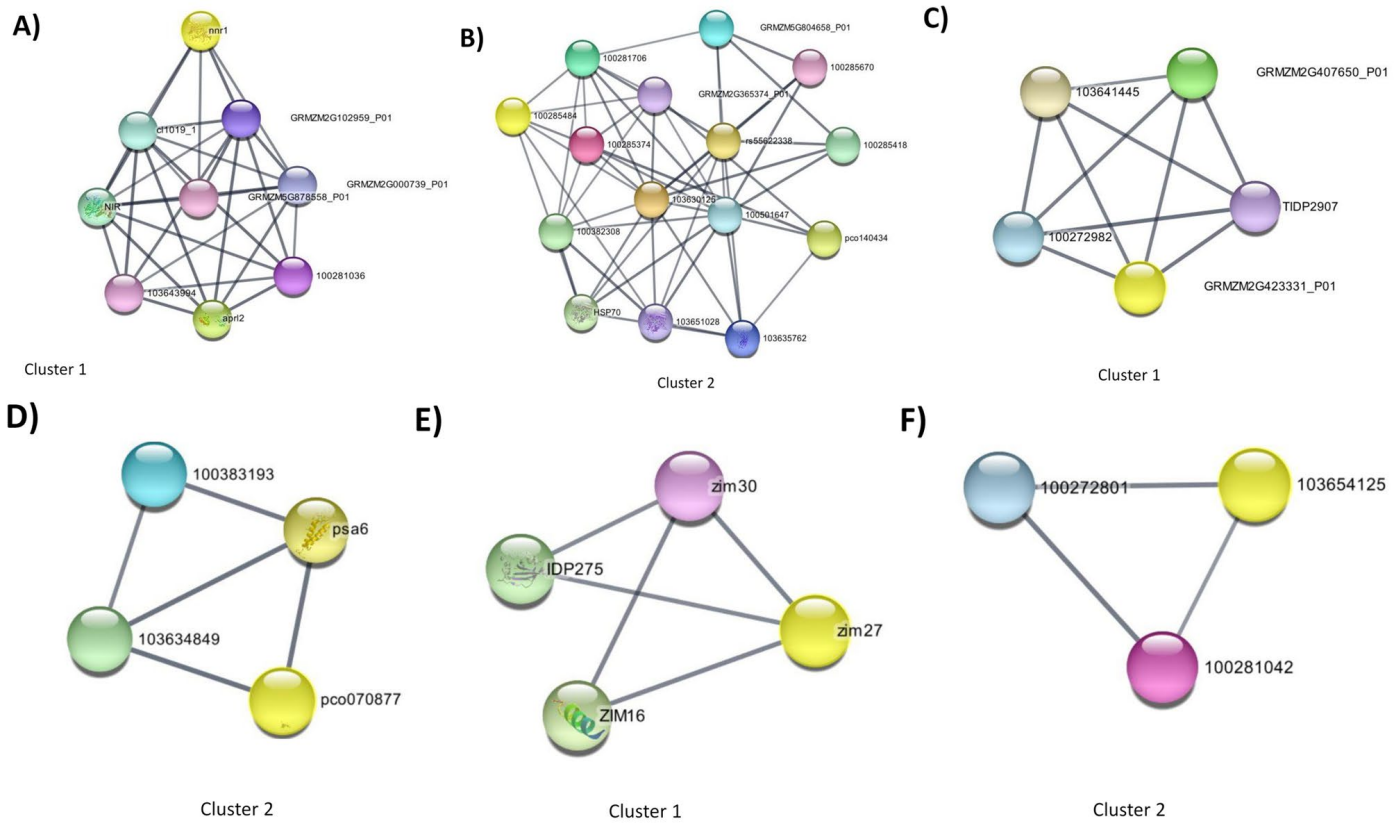
Network analysis of DEGs created using StringApp through the Cytoscape user interface. Two core clusters analyzed by MCODE for each type of stress are indicated above. **A** and **B** (core clusters from abiotic DEGs), C and D (core clusters from biotic DEGs), and E and F (core clusters from co-DEGs): The node colors are arbitrary, and the words prefaced by “GRMZM” are associated with B73 version 3 gene models; these are the STRINGdb identifiers for the maize proteins. StringApp uses the maize reference genome B73 version 3 (RefGen_V3). However, we have included the corresponding latest version; B73v5 for each of the B73v3 gene models in our table. (Additional file 6). The numbers indicated on the nodes are gene IDs (linked to NCBI), the numbers are displayed for genes without a gene symbol in the network. More details of the Clusters for each stress type with phylostrata scores is indicated in Additional file 6

Interactions and identification of hub genes from the abiotic DEGs were generated using MCODE (Methods). For this group of genes, high-ranked clusters were analyzed (Additional file 6). The most recent common ancestor (mrca) and phylostrata values generated by PhylostratR (Methods) were assigned to the genes in each of the clusters, together with the gene description and UniProt accessions (Additional file 6). The network analysis using STRING helps to identify connections among DEGs common to abiotic stress. The hub genes for core clusters 1 and 2 can be seen in Fig. 5A **and B.** The clusters reveal a close functional relationship between the proteins or genes involve in abiotic stress. For example, Cluster 1 (Fig. 5A) comprises nine genes: nnr1 (*Zm0001EB17670*), *cl1019*_1(*Zm00001eb166390*), *NIR* (*Zm00001eb193660*), *aprl2* (*Zm00001eb105800*), Zm*00001eb255880*, *Zm00001eb256260*, *Zm00001eb358930*, *Zm00001eb056910*, and *Zm00001eb089390* (Additional file 6). The GO biological process GO terms associated with this cluster include “nitric oxide biosynthesis process”, “sulfate assimilation” and “reactive nitrogen species metabolic process”. One of the molecular functions associated with genes in this Cluster is “adenylylsulfate kinase activity”, and they belong to the Sulfur and Purine metabolism pathway (Supplementary Fig. 1 of Additional file 7). All the genes in Cluster 1 were ancient genes with phylostrata score of 1 (cellular organisms). Cluster 2 (Fig. 5B, Additional file 6) comprises 15 genes, and 13 out of the 15 genes codes for heat shock proteins or chaperons. Twelve of the genes had a phylostrata score of 1 (cellular organisms) and 3 had a phylostrata score of 2 (eukaryote). The biological processes associated with Cluster 2 include “protein folding” and “cellular response to unfolded protein”. The molecular functions of genes in Cluster 2 include “heat shock protein binding” and “ATP-dependent protein folding chaperone”. The biological process associated with genes in Cluster 3 of abiotic DEG network is “rhythmic process” and is enriched in the circadian rhythm pathway (Supplementary Fig. 3 of Additional file 7). With the exception of Clusters 8 and 17, all the core clusters from the abiotic DEGs contained genes with more ancient phylostrata, and genes with a similar phylostrata score clustered together.

#### Interaction network of biotic stress genes and phylostratigraphic analysis

Interactions and identification of hub genes from the biotic DEGs were generated using MCODE (Methods). The network analysis revealed clusters described in Additional file 6. Cluster 1 (Fig. 5C) was made up of five genes, all within phylostrata 1-cellular organisms. Cluster 2 (Fig. 5D) contained four genes, falling within phylostrata 3-Viridiplantae, or 4-Streptophyta). The genes of cluster 2 include: *psa6* (*Zm00001eb29999000*), *pco070877* (*Zm00001eb1517500*), *Zm00001eb346280* and *Zm00001eb336410*. The biological process associated with the genes of this Cluster is “photosynthesis” (Supplementary Fig. 6 of Additional file 7). We found that the genes in Cluster 2 were generally down-regulated. Cluster 3 had three genes that fell within phylostrata 1-cellular organism and 4-Streptophyta. The biological process associated with hub genes of Cluster 3 is “Glutathione metabolic process” (Supplementary Fig. 7 of Additional file 7). Cluster 4 comprised of three genes within phylostrata 6-Embryophyta, and8-Euphyllophyta. Similar to the abiotic DEGs, biotic DEGs with similar phylostrata from the network analysis clustered together, but unlike the abiotic DEGs, the biotic DEGs showed a more diverse phylostrata profile.

### Interaction network of co-DEGs and phylostratigraphic analysis

We investigated the interaction of the overlapping genes to find the hub of genes among the co-DEGs. We found only two clusters from the network analysis of the co-DEGs (cluster analysis summarized in Additional file 6). The two clusters are indicated in Fig. 5E **& F**. Cluster 1 was made up of four genes (phylostrata 6-Embryophyata, 7-Tracheophyta, 11-Mesangiospermae). These hub genes include for Cluster 1 are *Zm00001eb369060* (IDP275, putative NAC domain transcription factor superfamily protein; Uncharacterized protein), *Zm00001eb223590* (zim30), *Zm00001eb006000* (Zim16, ZIM motif family protein), *Zm00001eb005990* (zim27, uncharacterized). Cluster 2 contained three genes (all genes had a phylostrata of 1-cellular organisms) and the hub genes for Cluster 2 were: *Zm00001eb346150* (Putative cytochrome P450 superfamily protein, uncharacterized), *Zm00001eb191610* (Cinnamoyl-CoA reductase 1), *Zm00001eb077220* (Phenylalanine ammonia-lyase). Similar to abiotic and biotic DEGs, the seven hub genes of the co-DEGs clustering together shared similar phylostrata. To explore the molecular mechanism of the hub genes identified here, we performed enrichment analysis of genes from the clusters. Hub genes from Cluster 1 were associated with biological processes; “regulation of jasmonic acid-mediated signaling pathway”, “response to wounding, and “regulation of defense response”. Genes from Cluster 2 were significantly enriched in the GO terms “phenylpropanoid metabolic process”, “secondary metabolic process”, “lignin metabolic process”, and “phenylpropanoid biosynthesis pathway” (Supplementary Figs. 8 and 9 of Additional file 7).

## Discussion

Under natural conditions, plants are exposed to a combination of abiotic and biotic stresses, and to withstand these stresses, plants respond with a series of changes in their transcriptome. Research has shown that studying gene clusters rather than individual genes is more effective in understanding plant stress tolerance [23]. Therefore, meta-analysis of different transcriptome data has been found as a robust approach to studying multiple stress tolerance in plants. While meta-analysis of RNA-Seq in maize induced by various fungal pathogens has been performed previously [18] and similar meta-analysis under abiotic stress conditions revealed DEGs regulated by abiotic stress in cotton [23], Arabidopsis [24] and wheat [25], our study appears to be the first to explore candidate genes using meta-analysis of publicly available maize biotic and abiotic transcriptome data.

With our meta-analysis using 24 publicly available RNA-Seq datasets of maize under various stresses, we identified 2,555 and 408 abiotic and biotic regulated genes (DEGs), respectively. We also identified 267 co-DEGs in response to all the stresses mentioned in this study. While the fact that more abiotic than biotic DEGs were observed might be due to fewer available B73 abiotic vs. biotic RNA-Seq experiment studies, meta-analysis of rice transcriptome data (five publicly available abiotic stress and six biotic stress experiments) identified 5,863 and 2,154 genes that are differentially regulated by abiotic and biotic stress respectively [26], suggesting that there might be fewer biotic DEGs generally among plants. Additionally, a meta-analysis of tomato using 213 abiotic and 178 biotic microarray samples found 1,862 and 835 abiotic and biotic regulated genes, respectively [23].

Late Embryogenesis Abundance (LEA) proteins are known to be involved in plant response to abiotic stresses such as drought, cold, and salinity. From our analysis, LEA proteins were only found in the abiotic DEGs, and this is consistent with previous reports in maize and wheat [27, 28]. Heat-shock Proteins (HSPs) are involved in protein folding, activation, and transport, protecting proteins from degrading during stress [27], and we identified HSPs from abiotic, biotic and overlapping DEGs, confirming the importance of HSPs in regulating all types of stress. HSPs’ response to stress depends on the intensity and varying length of stress, and this could account for the many HSPs identified in response to abiotic stress compared to biotic stress. However, evidence of HSPs in biotic stress tolerance has been elaborated [29].

The candidate DEGs that were significantly induced from the biotic DEG analysis include potential PRR genes such as WAK-RLK (*Zm00001eb177830*), which is known to confer pathogen resistance in maize [18]. Other WAK-RLK genes (*Zm00001eb334620* and *Zm00001eb156230*) previously reported [18] were also identified in our biotic DEG list. Two LRR-RLK genes (*Zm00001eb293660* and *Zm00001eb153630*) identified as hub genes in the co-expression network of the meta-analysis study in maize during multiple pathogen stresses were identified in our list of biotic regulated DEGs, supporting the critical role that these genes would play in biotic stress response. Moreover, genes encoding Peroxidase (*Zm00001eb140320*) and P450 (*Zm00001eb043620*) previously reported as co-DEGs of multiple pathogen responses, were also found in our biotic DEG list; previous research indicates the role of several P450 genes in disease resistance in rice and barley [18, 30–32]. Additionally, two chitinase genes identified from the same report (*Zm00001eb167720* and *Zm00001eb167710*) believed to play a role in disease resistance in maize were found in our DEG list. However, *Zm00001eb167710* was also identified in our co-DEG list. There is evidence to support the role of chitinase in drought and heat stress in addition to been significantly induced during pathogen response. This could explain the presence of *Zm00001eb167710* among our co-DEGs [33, 34]. Interestingly, a salicylic acid (SA) marker gene *Zm00001eb032600* (*ZmPR5*) induced by different pathogens was also identified in our biotic regulated genes [18]. Comprehensive evidence of the role of SA in pathogenesis-related (PR) gene expression, systemic acquired resistance, and hypersensitive response has been reported, although it regulates some abiotic stresses [35, 36]. The co-DEGs (267 genes) identified in this work are hypothesized to be involved in a cross-talk between abiotic and biotic stress response. A gene encoding ATP binding cassette (ABC) transporter (*Zm00001eb357950*) was validated in response to three pathogens. Although this gene was induced by biotic stress from the previous study [18] we found it in our co-DEG list after the meta-analysis.

Transcription factors are key regulators of plant growth and development, and could play essential roles in regulatory networks to improve abiotic and biotic stress tolerance in plants [37]. Major TF plant families such as EREB, MYB, WRKY, bHLH, bZIP, NAC, and GRAS were differentially expressed TFs from our analysis. Major TF families such as NAC, AP2/ERF, bZIP and MYB have been reported as key regulators in plant responses to biotic and abiotic stress [9, 38, 39]. The EREB TF family was the largest of the TFs differentially regulated by abiotic stress from our analysis. To support our finding, we found that the ERF TF family was the largest TF family identified in seedling maize in response to abiotic stress [9]. Interestingly, most of the differentially expressed TFs from the biotic DEGs were from the WRKY TF family. These TFs, WRKY64, (*Zm00001eb159340*), WRKY115 (*Zm00001eb368640*), and WRKY108 (*Zm00001eb112840*) were also reported previously in [18]. *ZmWRKY83* (*Zm00001eb286490*) has previously been identified to be induced during *F. graminearum* infection [40]. From our analysis, *ZmWRKY83* was up-regulated in response to *F. venenatum* and Mites herbivores (both BGM and TSM). These findings support the significant role that *ZmWRKY83* could play in general pathogen response. Our findings suggest a role for *ZmWRKY40* in response to abiotic (cold and salinity) and biotic stress (Mites herbivores). Previous reports indicate that *ZmWRKY40* was induced by high salinity, drought, ABA, and high temperature [4]. Additionally, important role of *WRKY40* in PAMP-triggered basal defense in Malus has been outlined [41].

We found that the highest TF enrichment was in salt stress. The role of these TF families (EREB, NAC, MYB, WRKY, bHLH and bZIP) in salt tolerance have been reported [42]. Consistent with our findings of the TFs in response to salt stress, Lv et al. 2016 [43] noted a sharp response to 10 NAC TFs from watermelon (*Citrullus lanatus*) in response to salt stress. NACs response to salt treatment could suggest their probable role in plant salt stress tolerance. We also provide evidence to support the regulation of the TFs in drought; some NAC genes were found to be down-regulated after PEG treatment in *Citrullus lanatus* [43]. This could explain the number of down-regulated NAC TFs in our findings during drought stress (Table 1) suggesting the potential involvement of NAC in drought stress. It is worth mentioning that a putative NAC domain transcription factor superfamily protein (*Zm00001eb369060*) IDP275, a hub gene from our co-DEG list, was induced during combined stress of herbivory and drought stress [10]. From our analysis, this putative NAC TF was up-regulated under the abiotic stresses cold, heat, drought, salinity, cadmium, phosphorus, waterlogging, and ammonium. Also, this TF was up-regulated after infection with *Fusarium venenatum*, *Fusarium graminearum*, *Mites herbivores*-BGM infestation and *Colletotrichum graminicola*. In addition, we identified *ZmNAC126* among the co-DEGs. The role of *ZmNAC126* in chlorophyll degradation to enhance leaf senescence has been previously reported [44]. We hypothesize that the candidate genes identified here, especially the co-DEGs, could be regulators of multiple stress response in maize. Maize GATA transcription factors involved in environmental stress response, which were recently characterized in response to abiotic stress, were identified from the list of the abiotic DEGs from this study. These GATA TFs are *Zm00001eb240100* (putative GATA transcription factor 22, ZmGATA12), *Zm00001eb258290* (*ZmGATA14*), and *Zm00001eb385190* (*ZmGATA34*) [45]. Although these GATA TFs were found in response to some biotic stress from the report, we only found them to be regulated in our abiotic stresses. These findings could be due to GATA regulation by specific biotic stress conditions.

Many of our results are supported by findings from previous studies, suggesting that our stress-response functional annotation pipeline is robust and accurate. For example, our functional annotation of biotic, abiotic and shared DEGs were significantly enriched in biological processes such as “response to stress”, “response to stimulus”, “response to external stimulus”, “response to salicylic acid mediated pathway”, “‘jasmonic acid mediated pathway”, and “abscisic acid-activated signaling pathway” (Fig. 3, Additional file 3, Additional file 8, and Additional file 9). These terms were also among the enriched GO terms in both biotic and abiotic DEGs in tomatoes and rice, supporting the role of hormone and metabolism as part of plant stress adaptation [23, 26]. Also, “secondary metabolic process” was one of the most enriched GO terms among the biotic, abiotic and co-DEGs, and our KEGG pathway analysis showed that DEGs from biotic, abiotic and the co-DEGs were significantly enriched for “biosynthesis of secondary metabolites”, which is consistent with previous report by Tahmasebi et al., 2019 [23].

We performed phylostratigraphic analyses on abiotic, biotic, and co-DEGs of each of the core genes from our cluster analyses. Although some of the clusters had genes with different phylostrata, for most of the clusters, genes with the same phylostrata clustered together. From our analysis, most of the abiotic stress genes belonged to the lowest a phylostrata of 1, or cellular organisms, suggesting an ancient origin. Phylostratigraphic analysis in Arabidopsis during abiotic stress revealed a considerable number of abiotic stress genes with ancient origin as well, and that genes of the same age tend to link together in a stress gene network [21]. Similarly, using a phylotranscriptomic approach, the average gene age and divergence of induced genes was identified in response to biotic stress (herbivore elicitation) in tobacco [46]. Understanding of the evolution of stress genes could show the tempo of plant adaptation to stress and support discovery and functional characterization of stress-related genes [21]. Notably, genes from cluster 8 and 17 of the abiotic DEGs in our analysis were made up of genes with somewhat higher phylostrata, or of more recent origin (Additional file 6). The difference in the ages of genes within a network could be as a result of changes in the composition of genes of different abiotic stress. Also, genes from these clusters could be from different types of abiotic stress. In the process of evolution, new genes could be introduced to affect the function of a group of genes, and this reason could explain how abiotic stress genes could have higher phylostrata than biotic genes. Another potential reason for abiotic genes belonging to higher phylostrata is that, in the natural environment, stress factors occur concurrently or in combination, and plants develop shared responses [21].

We investigated the GO terms associated with the hub genes of the clusters. We found that the biological processes “nitric oxide biosynthesis”, “protein folding” and “rhythmic process” are key processes involved in abiotic stress tolerance in plants. The biological process “nitric oxide biosynthesis” enriched by genes from Cluster 1 of the abiotic DEGs has been reported in response to drought, salinity, oxidative and heavy metal stress [47]. The significance of “protein folding” by heat shock factors in regulating different stresses have been elaborated [21, 29]. Additionally, strong evidence of the importance of the “rhythmic process” in controlling different types of abiotic stress-responsive genes have been discussed [48]. Moreover, we identified “photosynthesis” as a biological process of genes belonging to Cluster 2 of the biotic DEGs. We further identified that all the genes in this cluster were down-regulated. This result supports previous findings of rice generally down-regulating photosynthesis during biotic and abiotic stress. It confirms the importance of the photosynthetic machinery in environmental stress response. Also, we found the biological process “Glutathione metabolic process” associated with hub genes of Cluster 3 of the biotic DEGs. Glutathione is mentioned as a crucial metabolite in the life of plants. Glutathione metabolism is reported as one of the most ancient defense systems in plants and regulates abiotic and biotic stresses [49, 50]. Our findings of co-DEG hub genes in the regulation of Jasmonic acid mediated signaling pathway is consistent with report of stress-induced hormone-responsive genes in rice [26]. Hub genes in this cluster were mostly up-regulated in our analysis in both biotic and abiotic stress. The phenylpropanoid metabolic process was enriched in the hub genes of cluster 2 of the co-DEGs. Genes from the phenylpropanoid pathway, such as phenylalanine ammonium lyases (PALs), are involved in lignin synthesis, reinforcing cell wall, and is important in plant immune defense system [18, 28]. Also, the phenylpropanoid pathway was significant during drought and heat stress in switchgrass, with PAL showing response to abiotic stresses [34, 51, 52].

## Conclusions

Our meta-analysis has revealed key genes, TFs, biological processes, and pathways regulated by abiotic stress, biotic stress and both stress types. Among the novel findings of this work, our analyses show that hormone-responsive and phenylpropanoid pathways are important in both combined, abiotic, and biotic stress response. We also found that the phylostrata of the hub genes of the same age in maize tend to be connected together in the network. The genes and TFs identified can further be characterized in maize to explore or establish their role in biotic and abiotic stress tolerance. The availability of 24 RNA-Seq datasets, all mapped using a standardized approach to the latest maize reference genome, offers a gold-standard dataset for in-depth exploration of the roles of gene expression in maize stress responses.

## Materials and methods

### Sample collection, data processing, and differential expression analysis

Twenty-four [24] high-quality RNA-Seq datasets from published RNA-Seq studies related to biotic and abiotic stress generated from tissues of the B73 cultivar were used in this analysis (Table 3). To be considered high quality, an expression dataset must minimally meet the following criteria: (1) published in a peer-reviewed journal; (2) deposited in a public data repository; (3) collected under controlled conditions with replicates; and (4) provided with metadata and a detailed method description. The dataset was downloaded in FASTQ format from the European Nucleotide Archive (ENA) server. The reads were mapped to the B73v5 reference genome (Zm-B73-REFERENCE-NAM-5.0) in January, 2023 using the STAR-2.7.2b program [53] followed by counting reads using the Subread package FeatureCounts [54]. This was followed by normalization and then determination of significant gene expression changes between controls and treated samples using the R program EdgeR [55]. DEGs were determined with a cut-off value [|log2(fold change) | ≥ 1, *p*-value < 0.05]. Meta-analysis of abiotic and biotic stress was performed separately to identify DEGs involved in both stress conditions. To visualize the unique and overlap of significantly DEGs (*p* < 0.05) of abiotic and biotic stress-responsive genes, a Venn-diagram was used (VENNY v.2.1 https://bioinfogp.cnb.csic.es/tools/venny/index.html). The methods developed to map the high-quality RNA-Seq reads have been illustrated in Fig. 1. The pipeline was validated by bench-marking fold-change values from the pipeline against published fold-change values (Additional file 10). The list of DEGs were uploaded to MaizeMine (a data mining resource) to obtain a description/annotation of the DEGs [56].

**Table 3:** Summary of RNA-Seq reads from projects mapped to the latest maize reference genome B73v5. The details of each project are indicated in Additional file

Project Publication Year	Number of Publications/ Projects	Publicly available Ref Genome at Year of Publication	Release Date	Ref Genome used for Mapped Reads
2014	2	B73 RefGen_v3	2013	B73 RefGen_v2
2015	2	B73 RefGen_v3	2013	B73 RefGen_v2
2016	2	Zm-B73-REFERENCE-GRAMENE-4.0	2016	B73 RefGen_v3
2017	4	Zm-B73-REFERENCE-GRAMENE-4.0	2016	B73 RefGen_v3
2018	2	Zm-B73-REFERENCE-GRAMENE-4.0	2016	B73 RefGen_v2
2018	1	Zm-B73-REFERENCE-GRAMENE-4.0	2016	B73 RefGen_v3
2019	1	Zm-B73-REFERENCE-NAM-5.0	2019	Zm-B73-REFERENCE-GRAMENE-4.0
2020	4	Zm-B73-REFERENCE-NAM-5.0	2019	Zm-B73-REFERENCE-GRAMENE-4.0
2021	3	Zm-B73-REFERENCE-NAM-5.0	2019	Zm-B73-REFERENCE-GRAMENE-4.0
2022	2	Zm-B73-REFERENCE-NAM-5.0	2019	Zm-B73-REFERENCE-GRAMENE-4.0
2022	1	Zm-B73-REFERENCE-NAM-5.0	2019	B73(de novo assembly)

### Functional analysis of stress responsive genes

#### Gene ontology enrichment analysis

The expressed stress-responsive genes were analyzed for enriched GO categories using Singular Enrichment Analysis (SEA) from AgriGO v2.0 (GO analysis toolkit and database for the agriculture community; http://systemsbiology.cau.edu.cn/agriGOv2/ with the maize reference genome B73 as the background (Maize AGPv4 (Maize-Gamer) [57]. The overrepresented terms in the three categories, biological process, cellular component, and molecular function were filtered based on the statistical information which is Fisher’s exact test and Bonferroni multi-test adjustment with *p*-value < 0.05. The number of input gene lists and the *p*-values were plotted against the GO terms (Fig. 3, Additional file 11).

#### KEGG pathway enrichment analysis

To provide further annotation to the DEGs we performed the Kyoto Encyclopedia of Genes and Genomes (KEGG) pathway enrichment analysis using ShinyGO v.0.77 [58] and g: Profiler online tool [59]. An adjusted *P*-value < 0.05 was used as a threshold for significantly enriched pathways.

### Network analysis and hub gene cluster identification

Gene networks for the set of abiotic, biotic and co-DEG differentially expressed genes were constructed with 0.7 level of confidence using STRINGApp [60]. Cytoscape v.3.9.1 was then used to visualize the presentation of the results from STRING. The Molecular Complex Detection (MCODE) Cytoscape plugin was further used to conduct hub genes cluster analysis [61]. Enrichment analysis for two clusters from each stress type was performed using g: Profiler to identify enriched GO terms and KEGG pathways associated with the clusters.

### Phylostratigraphic analysis

To provide additional functional characterization of the stress-responsive genes, we used a phylostratigraphic approach to extract the phylostratum of the DEGs using an R framework for Phylostratigraphy [62]. The strata 68,525.faa and 469,616.faa were pruned from the Uniprot data (obtained in April 2023) and Diamond [63] was run as a separate step. The step *focal_taxid* was set to 4577, *Zea mays*.

### Identification of transcription factors

The TFs annotated by GRASSIUS [64] used to show differential expression in response to abiotic and biotic stress were accessed from MaizeGDB [65]. We highlighted the number of the major TF families under various types of stress.

### Electronic supplementary material

Below is the link to the electronic supplementary material.

Supplementary Material 1

Supplementary Material 2

Supplementary Material 3

Supplementary Material 4

Supplementary Material 5

Supplementary Material 6

Supplementary Material 7

Supplementary Material 8

Supplementary Material 9

Supplementary Material 10

Supplementary Material 11

Supplementary Material 12

## Data Availability

All the stress-responsive genes identified together with their fold-change values from all the experiments and scripts used can be found at this link: https://ars-usda.app.box.com/v/maizegdb-public/folder/249920615860. These data are also hosted as JBrowse tracks at MaizeGDB (an example shown as Fig. 2B).

## Abbreviations

DEGs: Differentially Expressed Genes
GO: Gene Ontology
JA: Jasmonic acid
SA: Salicylic acid
ABA: Abscisic acid
KEGG: Kyoto Encyclopedia of Genes and Genome
GSR: Gibberella Stalk Rot
SCVM: Sugar Mosaic Virus
