# Supplementary material for: Functional annotation and meta-analysis of maize transcriptomes reveal genes involved in biotic and abiotic stress
Source: BMC Genomics. 2024 May 30;25:533. doi: 10.1186/s12864-024-10443-7 (PMC11137889; doi:10.1186/s12864-024-10443-7)
Supplement: Supplementary file 8 — Supplementary Material 8 [file 12864_2024_10443_MOESM8_ESM.pdf]

Supplementary data

Validating the pipeline to map the quality RNA-Seq reads

The pipeline was validated by having the fold-change values from the pipeline benchmarked against the published fold-change values. This is illustrated using one project accession. We selected heat stress (HT) verses normal temperature (NT) for the comparison

- NCBI BioProject Accession: PRJNA645274
- PudMed ID: 32756433 (doi: 10.3390/genes11080881)
- Publication year: 2020, reads previously mapped to B73v4
- Number of bioreps: 3
- Number of libraries: 42
- Reads mapped by MaizeGDB to Zm-B73-REFERENCE-NAM-5.0 (January, 2023)
- Stress type: Cold and heat stress (control and treatment groups)

Experimental conditions (all stress imposed for 2 hrs)

- Extreme low temperature (ELT) – 4 ^{O}C
- Medium Low temperature (MLT) – 10 ^{O}C
- Low temperature (LT) – 16 ^{O}C
- High temperature (HT) – 37 ^{O}C
- Medium High temperature (MHT) - 42 ^{O}C
- Extreme High temperature (EHT) – 48 ^{O}C
- Normal temperature (NT) – 25 ^{O}C

Supplementary Table 1. Compare the parameters from publication and our pipeline

A)

Parameters	Publication	Pipeline
DE analysis	DESeq2	EdgeR
Log2FC	$\vert \geq 1$	$\vert \geq 1$
P-value	< 0.05	< 0.05

B) Table to compare DEGs of selected stress conditions and number of DEGs using our pipeline

TS	DEGs from Publication	DEGs from Pipeline
ELT	9,166	9,307
MLT	9,235	9,493
LT	9,027	9,325
EHT	8,559	8,956

C)

Fold-change values of HTvsNT (high temperature vs normal temperature)

	Publication(B73v4)				Pipeline(B73v5)		
**GeneID**	**Log2FC**	**P-value**	**Annotation**		**GeneID**	**Log2FC**	**P-value**
Zm00001d022593	-10.33	3.86E-14	Diaminopimelate decarboxylase 2 chloroplastic		Zm00001eb331690	-9.33061	0.00038
Zm00001d011315	-4.75	5.07E-20	ABC transporter G family member 40		Zm00001eb357950	-5.58715	9.50E-05
Zm00001d042098	-4.22	6.02E-06	Glutathione S-transferase U16		Zm00001eb141030	-5.68969	9.05E-06
Zm00001d010360	-3.04	3.01E-19	Auxin-responsive protein IAA26		Zm00001eb350000	-3.9408	1.09E-05
Zm00001d025409	6.45	1.43E-05	Putative AP2/EREBP transcription factor superfamily protein		Zm00001eb422470	5.547858	0.0001731
Zm00001d012045	3.35	1.75E-05	Putative RING zinc finger domain superfamily protein		Zm00001eb364690	2.724765	0.0081347
Zm00001d043913	2.52	2.69E-06	Ethylene-responsive protein		Zm00001eb157180	3.251044	1.57E-05
Zm00001d024975	2.31	3.11E-05	Disease resistance protein RPM1		Zm00001eb419270	2.675773	3.06E-05
