# Supplementary material for: Functional annotation and meta-analysis of maize transcriptomes reveal genes involved in biotic and abiotic stress
Source: BMC Genomics. 2024 May 30;25:533. doi: 10.1186/s12864-024-10443-7 (PMC11137889; doi:10.1186/s12864-024-10443-7)
Supplement: Supplementary file 9 — Supplementary Material 9 [file 12864_2024_10443_MOESM9_ESM.pdf]

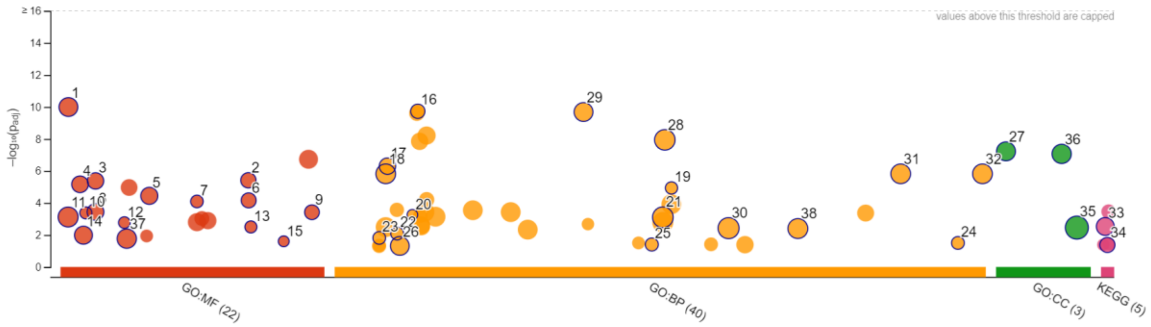

ID	Source	Term ID	Term Name	p_{adj} (query_1)
1	GO:MF	GO:0003700	DNA-binding transcription factor activity	1.014×10^{-10}
2	GO:MF	GO:0051082	unfolded protein binding	3.842×10^{-6}
3	GO:MF	GO:0005509	calcium ion binding	4.210×10^{-6}
4	GO:MF	GO:0004497	monooxygenase activity	6.819×10^{-6}
5	GO:MF	GO:0020037	heme binding	3.591×10^{-5}
6	GO:MF	GO:0051213	dioxygenase activity	6.898×10^{-5}
7	GO:MF	GO:0043621	protein self-association	8.052×10^{-5}
8	GO:MF	GO:0005506	iron ion binding	3.634×10^{-4}
9	GO:MF	GO:0140662	ATP-dependent protein folding chaperone	3.798×10^{-4}
10	GO:MF	GO:0004805	trehalose-phosphatase activity	4.155×10^{-4}
11	GO:MF	GO:0003677	DNA binding	7.555×10^{-4}
12	GO:MF	GO:0016160	amylase activity	1.687×10^{-3}
13	GO:MF	GO:0051787	misfolded protein binding	3.149×10^{-3}
14	GO:MF	GO:0004674	protein serine/threonine kinase activity	1.035×10^{-2}
15	GO:MF	GO:0102229	amylopectin maltohydrolase activity	2.481×10^{-2}
16	GO:BP	GO:0009408	response to heat	1.861×10^{-10}
17	GO:BP	GO:0006457	protein folding	4.990×10^{-7}
18	GO:BP	GO:0006355	regulation of DNA-templated transcription	1.512×10^{-6}
19	GO:BP	GO:0051259	protein complex oligomerization	1.154×10^{-5}
20	GO:BP	GO:0009065	glutamine family amino acid catabolic process	5.459×10^{-4}
21	GO:BP	GO:0050794	regulation of cellular process	7.781×10^{-4}
22	GO:BP	GO:0006986	response to unfolded protein	8.067×10^{-3}
23	GO:BP	GO:0005991	trehalose metabolic process	1.528×10^{-2}
24	GO:BP	GO:1990961	xenobiotic detoxification by transmembrane expor...	3.146×10^{-2}
25	GO:BP	GO:0048511	rhythmic process	3.933×10^{-2}
26	GO:BP	GO:0007154	cell communication	4.835×10^{-2}
27	GO:CC	GO:0005886	plasma membrane	5.965×10^{-8}
28	GO:BP	GO:0050896	response to stimulus	1.138×10^{-8}
29	GO:BP	GO:0042221	response to chemical	2.074×10^{-10}
30	GO:BP	GO:0065007	biological regulation	3.754×10^{-3}
31	GO:BP	GO:1903506	regulation of nucleic acid-templated transcription	1.512×10^{-6}
32	GO:BP	GO:2001141	regulation of RNA biosynthetic process	1.512×10^{-6}
33	KEGG	KEGG:01110	Biosynthesis of secondary metabolites	2.943×10^{-3}
34	KEGG	KEGG:04141	Protein processing in endoplasmic reticulum	4.203×10^{-2}
35	GO:CC	GO:0110165	cellular anatomical entity	3.482×10^{-3}
36	GO:CC	GO:0071944	cell periphery	8.587×10^{-8}
37	GO:MF	GO:0016491	oxidoreductase activity	1.693×10^{-2}
38	GO:BP	GO:0097659	nucleic acid-templated transcription	3.977×10^{-3}

version e109_eg56_p17_1d3191d
date 7/20/2023, 12:54:05 PM
organism zmays

g:Profiler

Supplementary figure 1. GO enrichment analysis of the abiotic DEGs using g: Profiler

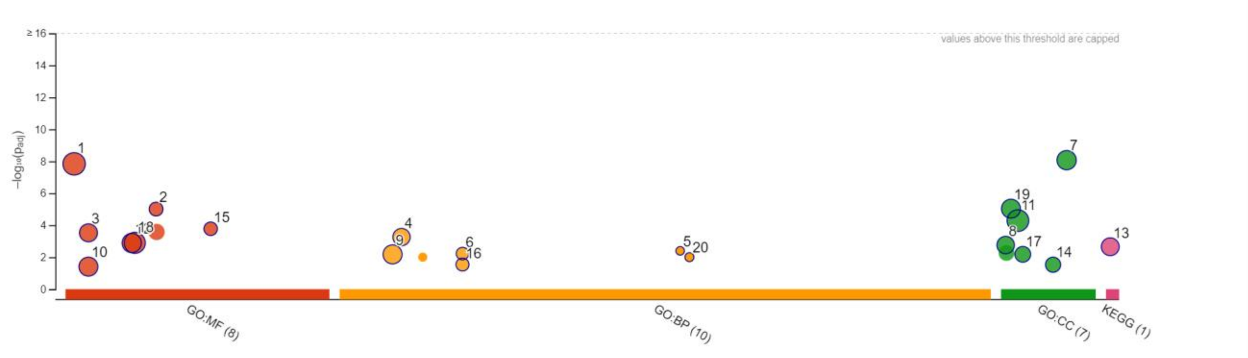

ID	Source	Term ID	Term Name	p_{adj} (query_1)
1	GO:MF	GO:0003824	catalytic activity	1.445×10^{-8}
2	GO:MF	GO:0030145	manganese ion binding	9.770×10^{-6}
3	GO:MF	GO:0004674	protein serine/threonine kinase activity	2.989×10^{-4}
4	GO:BP	GO:0006952	defense response	5.508×10^{-4}
5	GO:BP	GO:0051501	diterpene phytoalexin metabolic process	4.026×10^{-3}
6	GO:BP	GO:0016102	diterpenoid biosynthetic process	5.920×10^{-3}
7	GO:CC	GO:0071944	cell periphery	8.481×10^{-9}
8	GO:CC	GO:0005576	extracellular region	1.736×10^{-3}
9	GO:BP	GO:0006468	protein phosphorylation	6.685×10^{-3}
10	GO:MF	GO:0004672	protein kinase activity	3.891×10^{-2}
11	GO:CC	GO:0016020	membrane	5.134×10^{-5}
12	GO:MF	GO:0016491	oxidoreductase activity	1.287×10^{-3}
13	KEGG	KEGG:01110	Biosynthesis of secondary metabolites	2.216×10^{-3}
14	GO:CC	GO:0048046	apoplast	2.950×10^{-2}
15	GO:MF	GO:0045735	nutrient reservoir activity	1.663×10^{-4}
16	GO:BP	GO:0016101	diterpenoid metabolic process	2.876×10^{-2}
17	GO:CC	GO:0030312	external encapsulating structure	6.611×10^{-3}
18	GO:MF	GO:0016740	transferase activity	1.267×10^{-3}
19	GO:CC	GO:0005886	plasma membrane	9.211×10^{-6}
20	GO:BP	GO:0052315	phytoalexin biosynthetic process	9.977×10^{-3}

**version**	e109_eg56_p17_1d3191d
**date**	7/20/2023, 1:09:54 PM
**organism**	zmays

g:Profiler

Supplementary figure 2. GO enrichment analysis of the biotic DEGs using g: Profiler

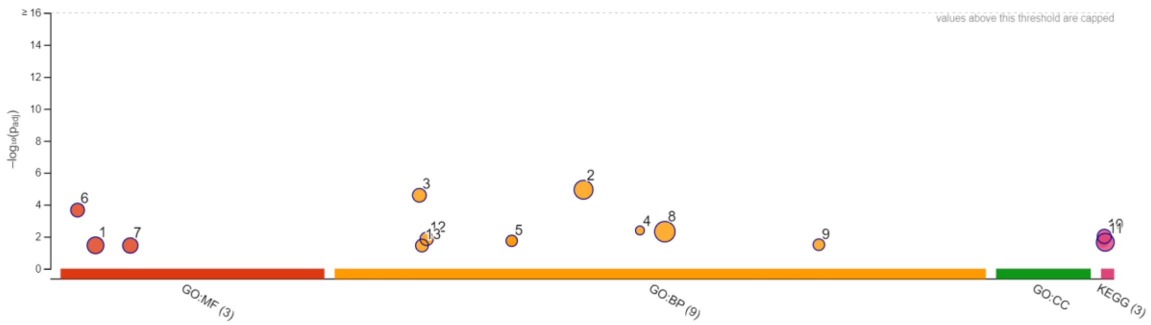

ID	Source	Term ID	Term Name	p_{adj} (query_1)
1	GO:MF	GO:0005506	iron ion binding	3.494×10^{-2}
2	GO:BP	GO:0042221	response to chemical	1.160×10^{-5}
3	GO:BP	GO:0009611	response to wounding	2.571×10^{-5}
4	GO:BP	GO:0046688	response to copper ion	4.086×10^{-3}
5	GO:BP	GO:0031408	oxylipin biosynthetic process	1.826×10^{-2}
6	GO:MF	GO:0004364	glutathione transferase activity	2.177×10^{-4}
7	GO:MF	GO:0016765	transferase activity, transferring alkyl or aryl (other...	3.590×10^{-2}
8	GO:BP	GO:0050896	response to stimulus	4.855×10^{-3}
9	GO:BP	GO:0120254	olefinic compound metabolic process	3.179×10^{-2}
10	KEGG	KEGG:00940	Phenylpropanoid biosynthesis	9.622×10^{-3}
11	KEGG	KEGG:01110	Biosynthesis of secondary metabolites	2.247×10^{-2}
12	GO:BP	GO:0010038	response to metal ion	1.366×10^{-2}
13	GO:BP	GO:0009751	response to salicylic acid	3.577×10^{-2}

version e109_eg56_p17_1d3191d
date 7/20/2023, 1:22:02 PM
organism zmays

g:Profiler

Supplementary figure 3. GO enrichment analysis of the co-DEGs using g: Profiler
