# Supplementary material for: Functional annotation and meta-analysis of maize transcriptomes reveal genes involved in biotic and abiotic stress
Source: BMC Genomics. 2024 May 30;25:533. doi: 10.1186/s12864-024-10443-7 (PMC11137889; doi:10.1186/s12864-024-10443-7)
Supplement: Supplementary file 10 — Supplementary Material 10 [file 12864_2024_10443_MOESM10_ESM.pdf]

Enrichment analysis of gene from the network analysis

Enrichment analysis of hub genes of the abiotic DEGs

Supplementary Fig.

Cluster 1

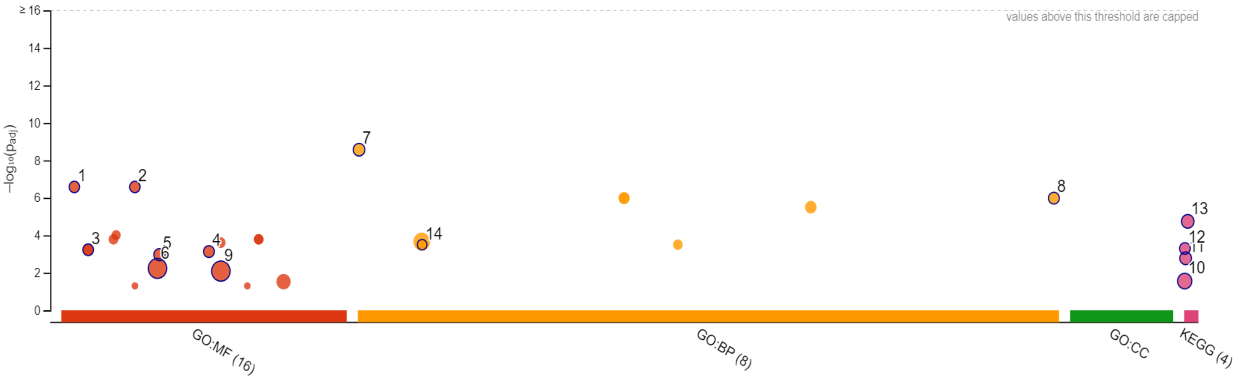

ID	Source	Term ID	Term Name	p_{adj} (query_1)
1	GO:MF	GO:0004020	adenylylsulfate kinase activity	2.626×10^{-7}
2	GO:MF	GO:0016661	oxidoreductase activity, acting on other nitrogeno...	2.626×10^{-7}
3	GO:MF	GO:0004779	sulfate adenylyltransferase activity	5.884×10^{-4}
4	GO:MF	GO:0043546	molybdopterin cofactor binding	7.354×10^{-4}
5	GO:MF	GO:0030151	molybdenum ion binding	1.078×10^{-3}
6	GO:MF	GO:0020037	heme binding	5.810×10^{-3}
7	GO:BP	GO:0000103	sulfate assimilation	2.700×10^{-9}
8	GO:BP	GO:2001057	reactive nitrogen species metabolic process	1.039×10^{-6}
9	GO:MF	GO:0046906	tetrapyrrole binding	8.108×10^{-3}
10	KEGG	KEGG:00230	Purine metabolism	2.762×10^{-2}
11	KEGG	KEGG:00450	Selenocompound metabolism	1.674×10^{-3}
12	KEGG	KEGG:00261	Monobactam biosynthesis	5.035×10^{-4}
13	KEGG	KEGG:00920	Sulfur metabolism	1.800×10^{-5}
14	GO:BP	GO:0006809	nitric oxide biosynthetic process	3.159×10^{-4}

**version**	e110_eg57_p18_4b54a898	g:Profiler
**date**	9/23/2023, 3:48:59 PM	
**organism**	zmays	

Supplementary Figure 1. Enrichment analysis of genes from cluster 1 of the abiotic DEGs. Cluster 1 comprised of nine genes

<u>**Supplementary Fig. 2**</u>

Cluster 2

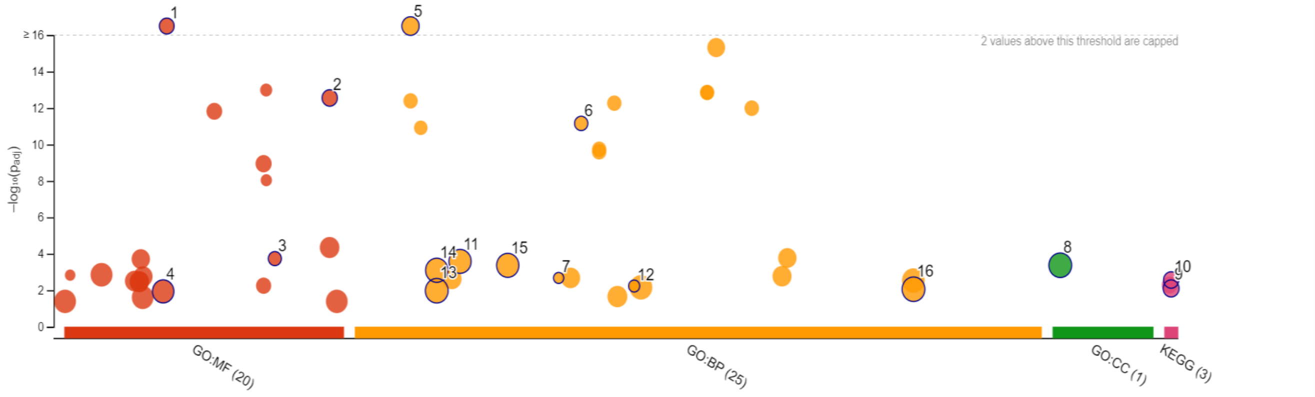

ID	Source	Term ID	Term Name	p_{adj} (query_1)
1	GO:MF	GO:0031072	heat shock protein binding	1.119×10^{-19}
2	GO:MF	GO:0140662	ATP-dependent protein folding chaperone	2.802×10^{-13}
3	GO:MF	GO:0060590	ATPase regulator activity	1.836×10^{-4}
4	GO:MF	GO:0030554	adenyl nucleotide binding	1.158×10^{-2}
5	GO:BP	GO:0006457	protein folding	2.126×10^{-17}
6	GO:BP	GO:0034620	cellular response to unfolded protein	6.989×10^{-12}
7	GO:BP	GO:0032781	positive regulation of ATP-dependent activity	2.113×10^{-3}
8	GO:CC	GO:0005737	cytoplasm	4.253×10^{-4}
9	KEGG	KEGG:04141	Protein processing in endoplasmic reticulum	7.978×10^{-3}
10	KEGG	KEGG:04144	Endocytosis	2.738×10^{-3}
11	GO:BP	GO:0010467	gene expression	2.601×10^{-4}
12	GO:BP	GO:0043462	regulation of ATP-dependent activity	5.877×10^{-3}
13	GO:BP	GO:0009058	biosynthetic process	1.062×10^{-2}
14	GO:BP	GO:0009059	macromolecule biosynthetic process	8.001×10^{-4}
15	GO:BP	GO:0019538	protein metabolic process	4.298×10^{-4}
16	GO:BP	GO:1901576	organic substance biosynthetic process	8.808×10^{-3}

**version**	e110_eg57_p18_4b54a898	g:Profiler
**date**	9/23/2023, 4:11:34 PM	
**organism**	zmays	

Supplementary Figure 2. Enrichment analysis of genes from cluster 2 of the abiotic DEGs. Cluster 2 comprised of 15 genes

<u>Supplementary Fig. 3</u>

Cluster 3

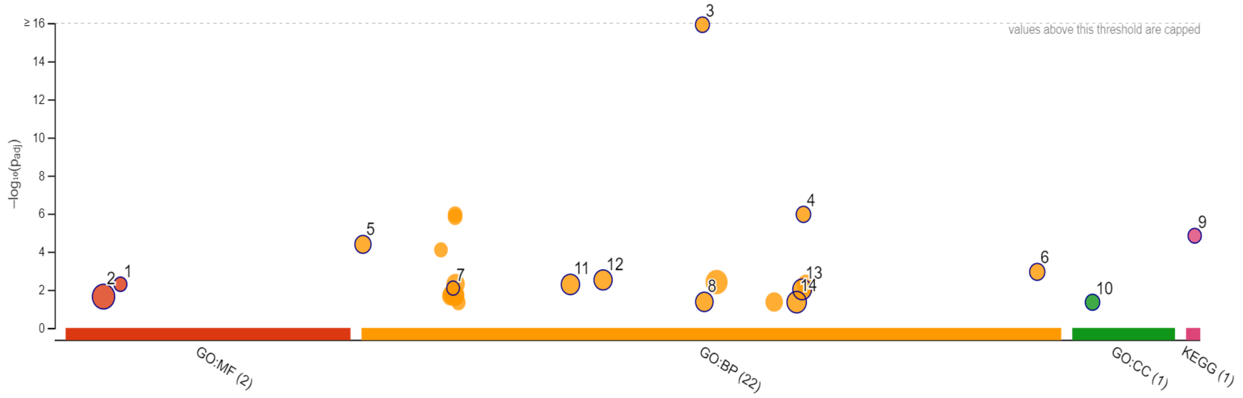

ID	Source	Term ID	Term Name	p_{adj} (query_1)
1	GO:MF	GO:0009881	photoreceptor activity	5.057×10^{-3}
2	GO:MF	GO:0005515	protein binding	2.258×10^{-2}
3	GO:BP	GO:0048511	rhythmic process	1.194×10^{-16}
4	GO:BP	GO:0071368	cellular response to cytokinin stimulus	1.083×10^{-6}
5	GO:BP	GO:0000160	phosphorelay signal transduction system	4.070×10^{-5}
6	GO:BP	GO:2000241	regulation of reproductive process	1.133×10^{-3}
7	GO:BP	GO:0009637	response to blue light	8.095×10^{-3}
8	GO:BP	GO:0048608	reproductive structure development	4.265×10^{-2}
9	KEGG	KEGG:04712	Circadian rhythm - plant	1.437×10^{-5}
10	GO:CC	GO:0019005	SCF ubiquitin ligase complex	4.461×10^{-2}
11	GO:BP	GO:0032870	cellular response to hormone stimulus	5.181×10^{-3}
12	GO:BP	GO:0035556	intracellular signal transduction	3.009×10^{-3}
13	GO:BP	GO:0071310	cellular response to organic substance	9.415×10^{-3}
14	GO:BP	GO:0070887	cellular response to chemical stimulus	4.410×10^{-2}

**version**	e110_eg57_p18_4b54a898	g:Profiler
**date**	9/23/2023, 4:19:13 PM	
**organism**	zmays	

Supplementary Figure 3. Enrichment analysis of genes from cluster 3 of the abiotic DEGs. Cluster 3 comprised of 8 genes

Supplementary Fig. 4

Cluster 4

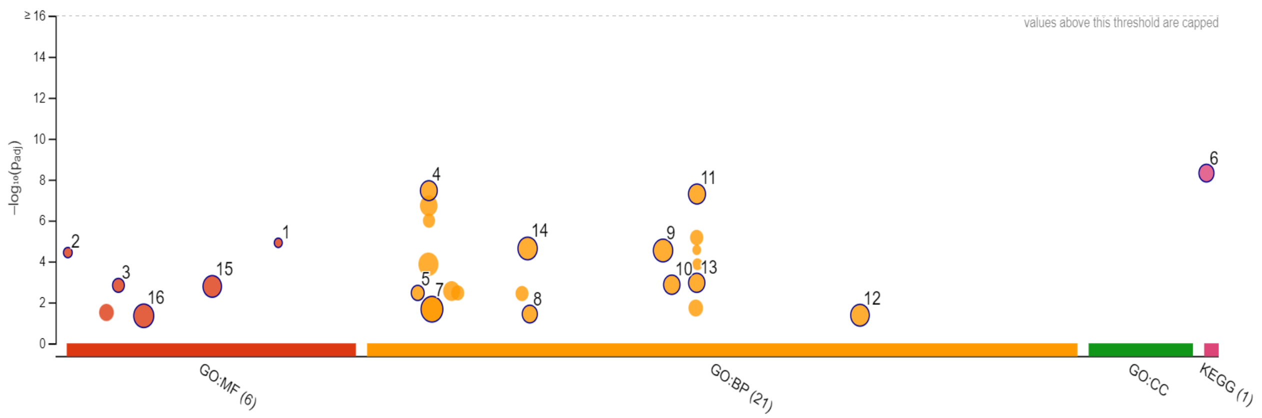

ID	Source	Term ID	Term Name	p_{adj} (query_1)
1	GO:MF	GO:0052667	phosphomethylethanolamine N-methyltransferase...	1.228×10^{-5}
2	GO:MF	GO:0000234	phosphoethanolamine N-methyltransferase activity	3.682×10^{-5}
3	GO:MF	GO:0008889	glycerophosphodiester phosphodiesterase activity	1.470×10^{-3}
4	GO:BP	GO:0006650	glycerophospholipid metabolic process	3.467×10^{-8}
5	GO:BP	GO:0006071	glycerol metabolic process	3.439×10^{-3}
6	KEGG	KEGG:00564	Glycerophospholipid metabolism	4.819×10^{-9}
7	GO:BP	GO:0006793	phosphorus metabolic process	2.156×10^{-2}
8	GO:BP	GO:0019751	polyol metabolic process	3.704×10^{-2}
9	GO:BP	GO:0044255	cellular lipid metabolic process	2.917×10^{-5}
10	GO:BP	GO:0045017	glycerolipid biosynthetic process	1.367×10^{-3}
11	GO:BP	GO:0046486	glycerolipid metabolic process	5.074×10^{-8}
12	GO:BP	GO:0090407	organophosphate biosynthetic process	4.235×10^{-2}
13	GO:BP	GO:0046474	glycerophospholipid biosynthetic process	1.104×10^{-3}
14	GO:BP	GO:0019637	organophosphate metabolic process	2.324×10^{-5}
15	GO:MF	GO:0042578	phosphoric ester hydrolase activity	1.660×10^{-3}
16	GO:MF	GO:0016788	hydrolase activity, acting on ester bonds	4.519×10^{-2}

**version**	e110_eg57_p18_4b54a898
**date**	9/23/2023, 4:25:41 PM
**organism**	zmays

g:Profiler

Supplementary Figure 4. Enrichment analysis of genes from cluster 4 of the abiotic DEGs. Cluster 4 comprised of 7 genes

Supplementary Fig. 5

Enrichment analysis of hub genes of the biotic DEGs

Cluster 1

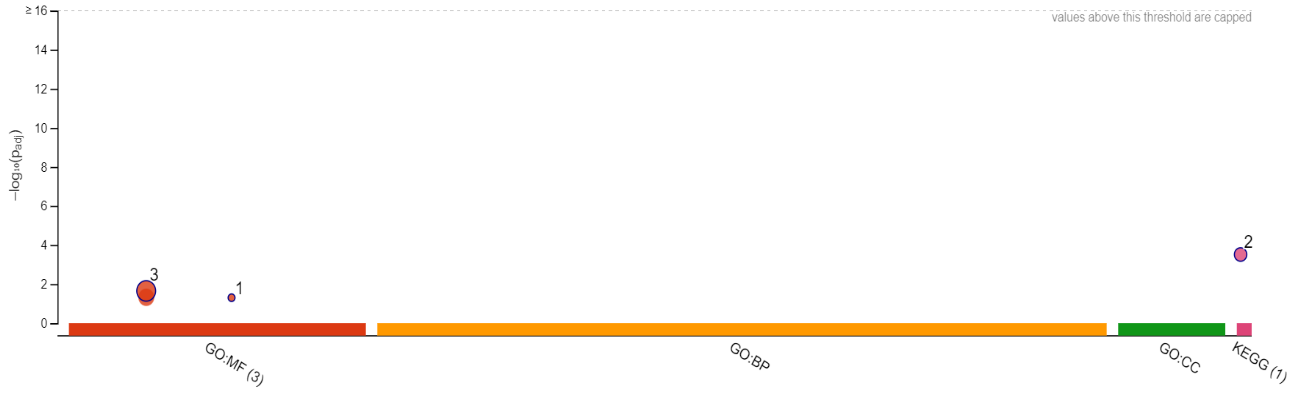

ID	Source	Term ID	Term Name	p_{adj} (query_1)
1	GO:MF	GO:0045486	naringenin 3-dioxygenase activity	4.995×10^{-2}
2	KEGG	KEGG:00941	Flavonoid biosynthesis	3.065×10^{-4}
3	GO:MF	GO:0016705	oxidoreductase activity, acting on paired donors, ...	2.243×10^{-2}

**version**	e110_eg57_p18_4b54a898
**date**	9/24/2023, 12:59:38 AM
**organism**	zmays

g:Profiler

Supplementary Figure 5. Enrichment analysis of genes from cluster 1 of the biotic DEGs. Cluster 1 of the network analysis of biotic DEGs comprised of 5 genes

Cluster 2

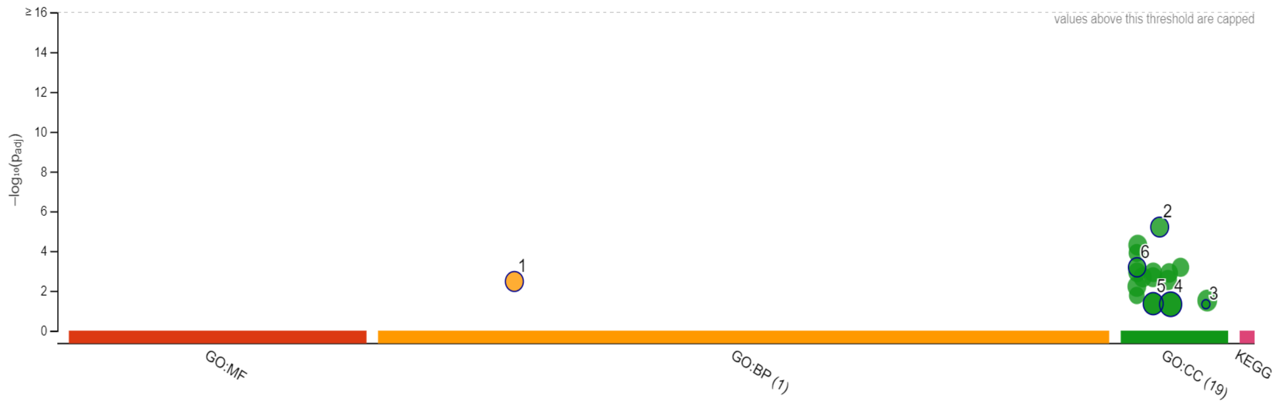

ID	Source	Term ID	Term Name	p_{adj} (query_1)
1	GO:BP	GO:0015979	photosynthesis	3.381×10^{-3}
2	GO:CC	GO:0034357	photosynthetic membrane	6.213×10^{-6}
3	GO:CC	GO:0098572	stromal side of plastid thylakoid membrane	4.704×10^{-2}
4	GO:CC	GO:0043228	non-membrane-bounded organelle	4.720×10^{-2}
5	GO:CC	GO:0031975	envelope	4.396×10^{-2}
6	GO:CC	GO:0009535	chloroplast thylakoid membrane	6.541×10^{-4}

**version**	e110_eg57_p18_4b54a898
**date**	9/24/2023, 1:03:24 AM
**organism**	zmays

g:Profiler

Supplementary Figure 6. Enrichment analysis of genes from cluster 2 of the biotic DEGs. Cluster 2 of the network analysis of biotic DEGs comprised of 4 genes

Supplementary Fig. 7

Cluster 3

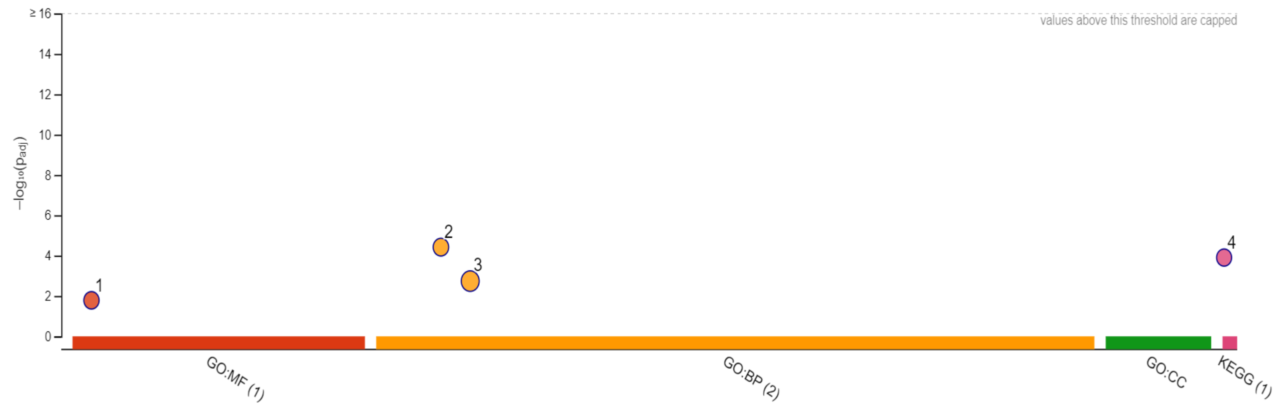

ID	Source	Term ID	Term Name	p_{adj} (query_1)
1	GO:MF	GO:0004364	glutathione transferase activity	1.635×10^{-2}
2	GO:BP	GO:0006749	glutathione metabolic process	3.779×10^{-5}
3	GO:BP	GO:0009636	response to toxic substance	1.824×10^{-3}
4	KEGG	KEGG:00480	Glutathione metabolism	1.244×10^{-4}

**version**	e110_eg57_p18_4b54a898
**date**	9/24/2023, 1:06:08 AM
**organism**	zmays

g:Profiler

Supplementary Figure 7. Enrichment analysis of genes from cluster 3 of the biotic DEGs. Cluster 3 of the network analysis of biotic DEGs comprised of 3 genes

<u>Supplementary Fig. 8</u>

Enrichment analysis of hub genes of the co-DEGs

Cluster 1

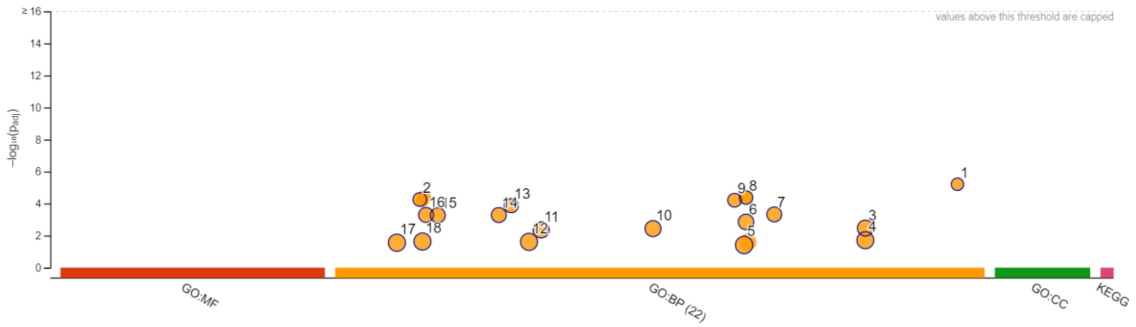

ID	Source	Term ID	Term Name	p_{adj} (query_1)
1	GO:BP	GO:2000022	regulation of jasmonic acid mediated signaling pathway	6.176×10^{-6}
2	GO:BP	GO:0009611	response to wounding	5.528×10^{-5}
3	GO:BP	GO:1901701	cellular response to oxygen-containing compound	3.381×10^{-3}
4	GO:BP	GO:1901700	response to oxygen-containing compound	1.944×10^{-2}
5	GO:BP	GO:0071310	cellular response to organic substance	3.845×10^{-2}
6	GO:BP	GO:0071396	cellular response to lipid	1.395×10^{-3}
7	GO:BP	GO:0080134	regulation of response to stress	4.745×10^{-4}
8	GO:BP	GO:0071398	cellular response to fatty acid	4.216×10^{-5}
9	GO:BP	GO:0070542	response to fatty acid	6.165×10^{-5}
10	GO:BP	GO:0048583	regulation of response to stimulus	3.640×10^{-3}
11	GO:BP	GO:0033993	response to lipid	4.382×10^{-3}
12	GO:BP	GO:0032870	cellular response to hormone stimulus	2.445×10^{-2}
13	GO:BP	GO:0031347	regulation of defense response	1.346×10^{-4}
14	GO:BP	GO:0023051	regulation of signaling	5.186×10^{-4}
15	GO:BP	GO:0010646	regulation of cell communication	5.370×10^{-4}
16	GO:BP	GO:0009966	regulation of signal transduction	5.096×10^{-4}
17	GO:BP	GO:0006952	defense response	2.776×10^{-2}
18	GO:BP	GO:0009755	hormone-mediated signaling pathway	2.341×10^{-2}

**version**	e110_eg57_p18_4b54a898
**date**	9/25/2023, 1:51:05 PM
**organism**	zmays

g:Profiler

Supplementary Figure 8. Enrichment analysis of genes from cluster 1 of the co-DEGs. Cluster 1 of the network analysis of the co-DEGs comprised of 4 genes

Supplementary Fig. 9

Cluster 2

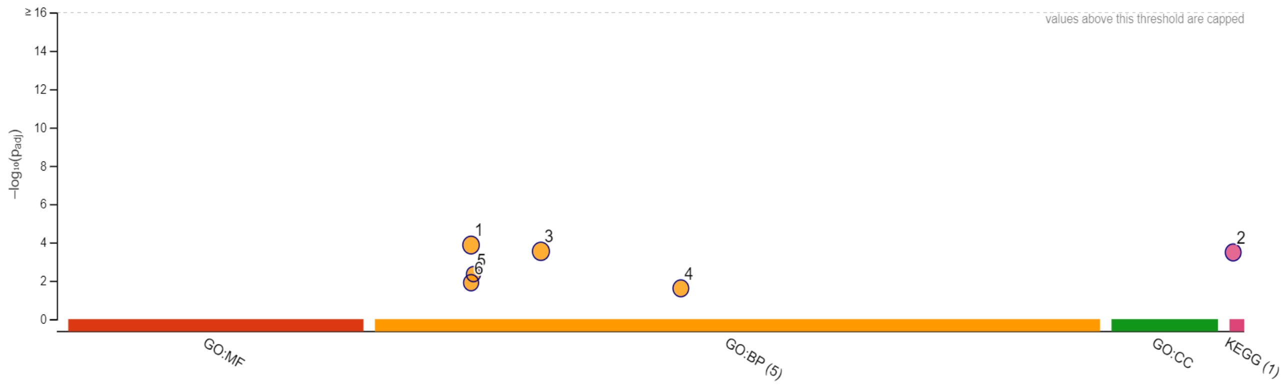

ID	Source	Term ID	Term Name	p_{adj} (query_1)
1	GO:BP	GO:0009698	phenylpropanoid metabolic process	1.364×10^{-4}
2	KEGG	KEGG:00940	Phenylpropanoid biosynthesis	3.301×10^{-4}
3	GO:BP	GO:0019748	secondary metabolic process	2.893×10^{-4}
4	GO:BP	GO:0044550	secondary metabolite biosynthetic process	2.471×10^{-2}
5	GO:BP	GO:0009808	lignin metabolic process	4.489×10^{-3}
6	GO:BP	GO:0009699	phenylpropanoid biosynthetic process	1.257×10^{-2}

**version**	e110_eg57_p18_4b54a898
**date**	9/24/2023, 1:16:36 AM
**organism**	zmays

g:Profiler

Supplementary Figure 9. Enrichment analysis of genes from cluster 2 of the co-DEGs. Cluster 2 of the network analysis of the co-DEGs comprised of 3 genes
