# Supplementary material for: Functional annotation and meta-analysis of maize transcriptomes reveal genes involved in biotic and abiotic stress
Source: BMC Genomics. 2024 May 30;25:533. doi: 10.1186/s12864-024-10443-7 (PMC11137889; doi:10.1186/s12864-024-10443-7)
Supplement: Supplementary file 11 — Supplementary Material 11 [file 12864_2024_10443_MOESM11_ESM.pdf]

Enriched GO terms for abiotic stress-responsive genes

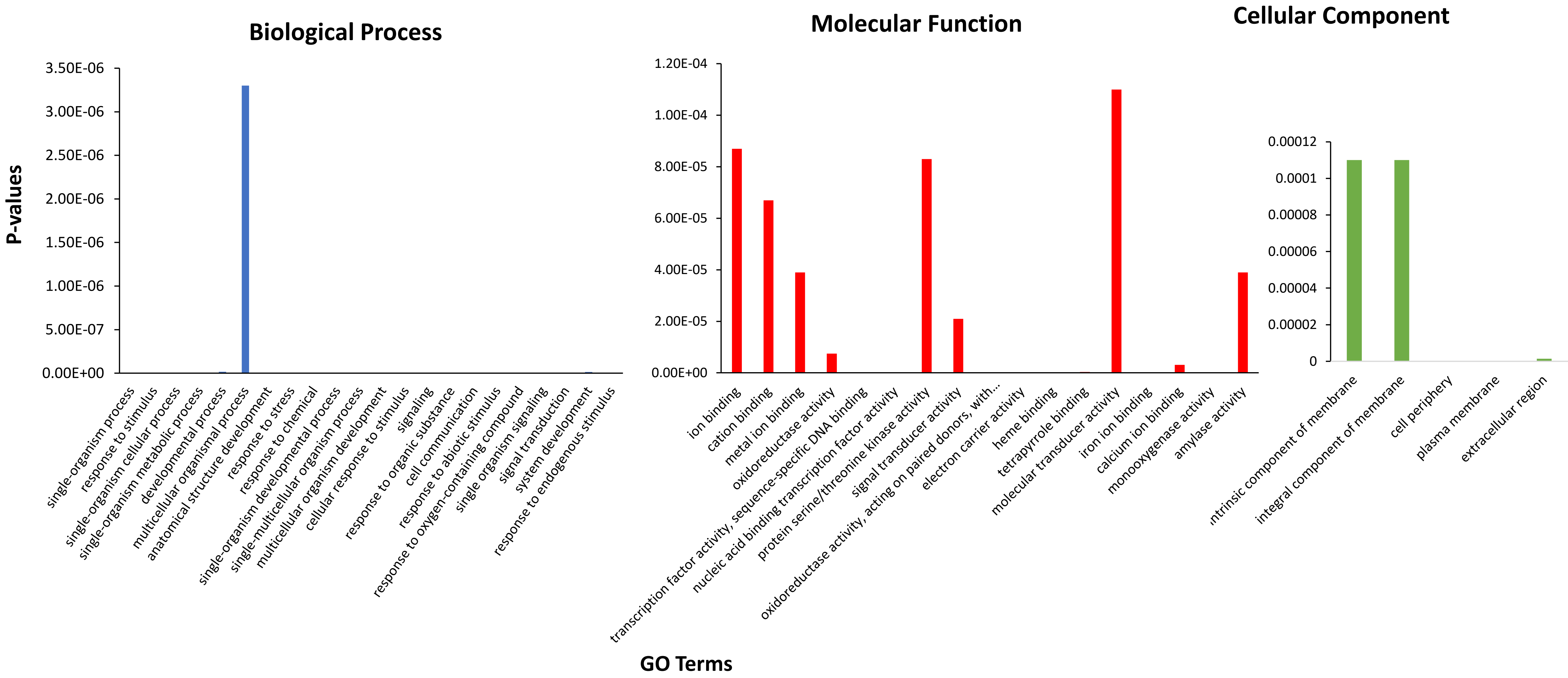

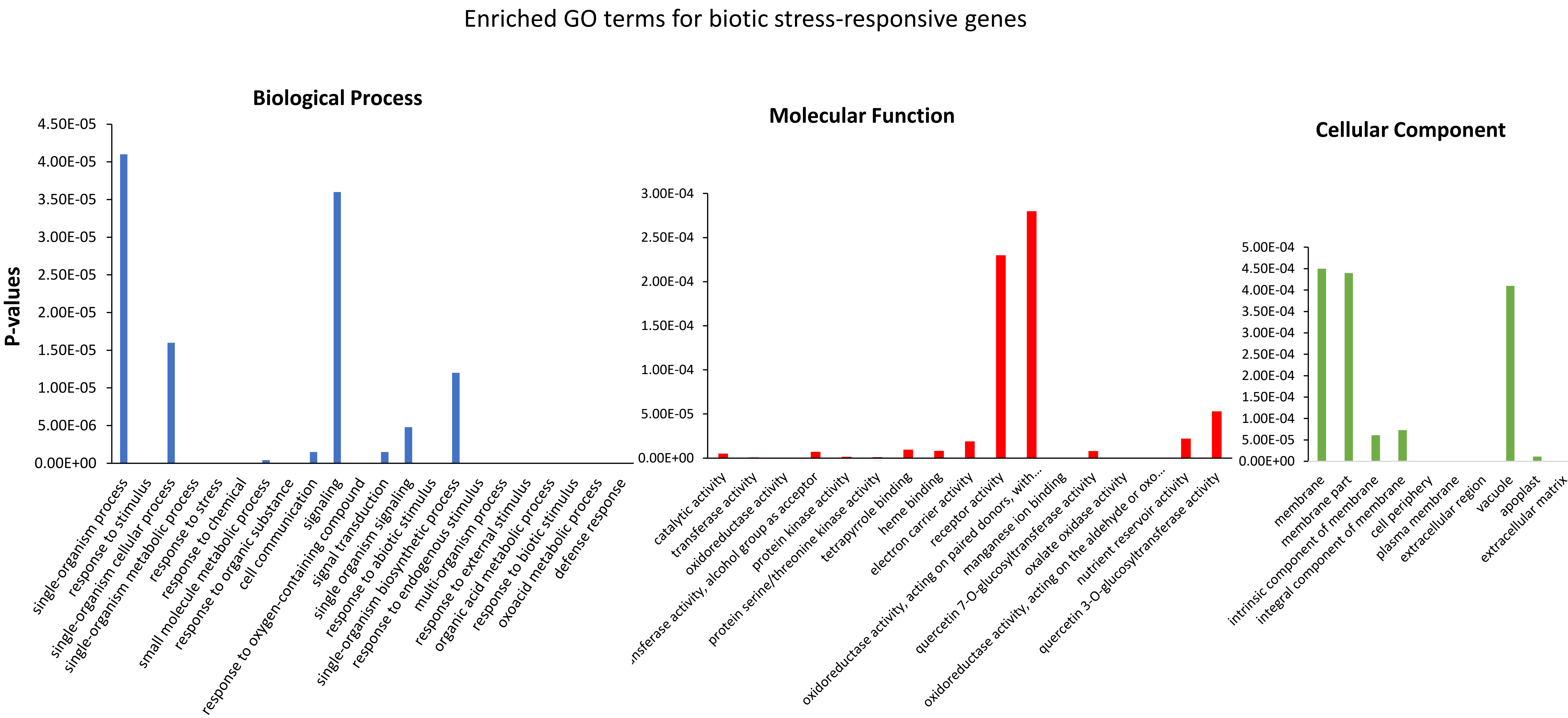

Enriched GO terms for biotic stress-responsive genes
Biological Process
4.50E-05
4.00E-05
3.50E-05
3.00E-05
2.50E-05
2.00E-05
1.50E-05
1.00E-05
5.00E-06
0.00E+00
P-values
single-organism process
response to stimulus
single-organism cellular process
single-organism metabolic process
response to stress
response to chemical
small molecule metabolic process
response to organic substance
cell communication
signaling
response to oxygen-containing compound
signal transduction
single organism signaling
response to abiotic stimulus
single-organism biosynthetic process
response to endogenous stimulus
multi-organism process
response to external stimulus
organic acid metabolic process
response to biotic stimulus
oxoacid metabolic process
defense response
Molecular Function
3.00E-04
2.50E-04
2.00E-04
1.50E-04
1.00E-04
5.00E-05
0.00E+00
catalytic activity
transferase activity
oxidoreductase activity
transferase activity, alcohol group as acceptor
protein kinase activity
protein serine/threonine kinase activity
tetrapyrrole binding
heme binding
electron carrier activity
receptor activity
oxidoreductase activity, acting on paired donors, with…
manganese ion binding
quercetin 7-O-glucosyltransferase activity
oxalate oxidase activity
oxidoreductase activity, acting on the aldehyde or oxo…
nutrient reservoir activity
quercetin 3-O-glucosyltransferase activity
Cellular Component
5.00E-04
4.50E-04
4.00E-04
3.50E-04
3.00E-04
2.50E-04
2.00E-04
1.50E-04
1.00E-04
5.00E-05
0.00E+00
membrane
membrane part
intrinsic component of membrane
integral component of membrane
cell periphery
plasma membrane
extracellular region
vacuole
apoplast
extracellular matrix
GO Terms

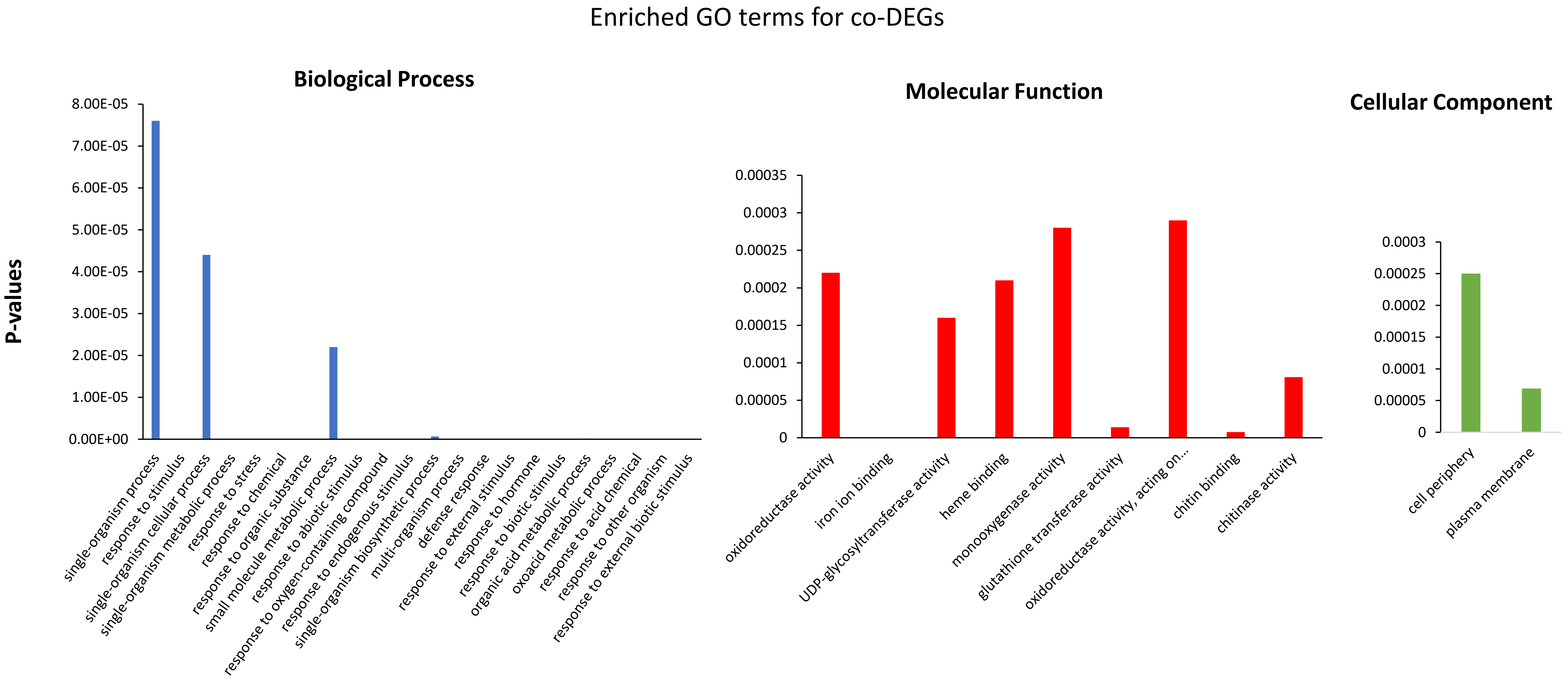

Enriched GO terms for co-DEGs
Biological Process
Molecular Function
Cellular Component
P-values
GO Terms
8.00E-05
7.00E-05
6.00E-05
5.00E-05
4.00E-05
3.00E-05
2.00E-05
1.00E-05
0.00E+00
single-organism process
response to stimulus
single-organism cellular process
single-organism metabolic process
response to stress
response to chemical
response to organic substance
small molecule metabolic process
response to abiotic stimulus
response to oxygen-containing compound
response to endogenous stimulus
single-organism biosynthetic process
multi-organism process
defense response
response to external stimulus
response to hormone
response to biotic stimulus
organic acid metabolic process
oxoacid metabolic process
response to acid chemical
response to other organism
response to external biotic stimulus
0.00035
0.0003
0.00025
0.0002
0.00015
0.0001
0.00005
0
oxidoreductase activity
iron ion binding
UDP-glycosyltransferase activity
heme binding
monooxygenase activity
glutathione transferase activity
oxidoreductase activity, acting on...
chitin binding
chitinase activity
0.0003
0.00025
0.0002
0.00015
0.0001
0.00005
0
cell periphery
plasma membrane

A) The Gene Ontology (GO) terms enriched by abiotic stress-responsive genes. B) GO terms enriched by biotic stress-responsive genes. C) GO terms enriched by co-DEGs. The GO terms are in the three GO domains (biological process, molecular function and cellular component). The P-value of each enriched term was plotted against the GO term
